# Improving the DNA specificity and applicability of base editing through protein engineering and protein delivery

**DOI:** 10.1038/ncomms15790

**Published:** 2017-06-06

**Authors:** Holly A. Rees, Alexis C. Komor, Wei-Hsi Yeh, Joana Caetano-Lopes, Matthew Warman, Albert S. B. Edge, David R. Liu

**Affiliations:** 1 Department of Chemistry and Chemical Biology, Harvard University, Cambridge, Massachusetts 02138, USA; 2 Howard Hughes Medical Institute, Harvard University, Cambridge, Massachusetts 02138, USA; 3 Broad Institute of MIT and Harvard, Cambridge, Massachusetts 02141, USA; 4 Eaton-Peabody Laboratory, Massachusetts Eye and Ear Infirmary, Boston, Massachusetts 02114, USA; 5 Program in Speech and Hearing Bioscience and Technology, Harvard Medical School, Boston, Massachusetts 02115, USA; 6 Orthopaedic Research Laboratories, Boston Children's Hospital, Boston, Massachusetts 02215, USA; 7 Department of Genetics, Harvard Medical School, Boston, Massachusetts 02215, USA; 8 Department of Otology and Laryngology, Harvard Medical School, Boston, Massachusetts 02115, USA

## Abstract

We recently developed base editing, a genome-editing approach that enables the programmable conversion of one base pair into another without double-stranded DNA cleavage, excess stochastic insertions and deletions, or dependence on homology-directed repair. The application of base editing is limited by off-target activity and reliance on intracellular DNA delivery. Here we describe two advances that address these limitations. First, we greatly reduce off-target base editing by installing mutations into our third-generation base editor (BE3) to generate a high-fidelity base editor (HF-BE3). Next, we purify and deliver BE3 and HF-BE3 as ribonucleoprotein (RNP) complexes into mammalian cells, establishing DNA-free base editing. RNP delivery of BE3 confers higher specificity even than plasmid transfection of HF-BE3, while maintaining comparable on-target editing levels. Finally, we apply these advances to deliver BE3 RNPs into both zebrafish embryos and the inner ear of live mice to achieve specific, DNA-free base editing *in vivo*.

## Abstract (web summary)

Third-generation base editors consist of a catalytically disabled Cas9 fused to a cytidine deaminase and a base excision repair inhibitor, enabling efficient, precise editing of individual base pairs in DNA. Here the authors describe engineering and protein delivery of base editors to improve their DNA specificity and enable specific base editing in live animals.

Traditional genome-editing agents introduce double-stranded DNA breaks as the first step of genome editing^1^,^2^,^3^,^4^. Cells respond to double-stranded DNA breaks primarily through non-homologous end joining, resulting in stochastic insertions or deletions (indels) at the cleavage site^1^,^5^. To generate more precise changes in genomic DNA, homology-directed repair (HDR) can be used to replace the genomic DNA surrounding the cleavage site with that of an exogenously supplied DNA donor template^6^,^7^,^8^. Unfortunately, HDR is typically accompanied by an excess of indels resulting from competing non-homologous end joining and is limited primarily to mitotic cells. In addition, most genome-editing methods rely on delivery of exogenous plasmid or viral DNA into mammalian cells followed by intracellular expression of the agent^9^,^10^,^11^,^12^. These delivery methods result in continuous, uncontrolled Cas9 and sgRNA expression even after the on-target locus has been edited, increasing the opportunity for genome editing at off-target loci^1^,^13^.

We recently described base editing, a different approach to genome editing that enables the direct, programmable, targeted conversion of a C:G base pair to a T:A base pair^3^,^14^. The third-generation base editor, BE3, contains in a single protein (i) a catalytically impaired Cas9 that opens a small single-stranded DNA bubble at a guide RNA-specified locus, (ii) a tethered single-strand specific cytidine deaminase that converts C to U within a window of approximately five nucleotides in the single-stranded DNA bubble, (iii) a uracil glycosylase inhibitor that inhibits base excision repair, thereby improving the efficiency and product selectivity of base editing and (iv) nickase activity to manipulate cellular mismatch repair into replacing the G-containing DNA strand. The combination of these components enables efficient and permanent C to T (or G to A) conversion in mammalian cells with minimal indel formation. Since our initial report^14^, other researchers have confirmed the ability of this strategy and related approaches to facilitate Cas9-directed C to T conversion in mammalian cells^15^,^16^,^17^ and in plants^18^.

Here we describe two advances that greatly improve the DNA specificity of base editing and that allow base editing *in vitro* and *in vivo* without supplying exogenous DNA, which has been associated with a risk of recombination with the host genome and cytotoxicity^18^,^19^. First, we engineer a mutant form of BE3 incorporating mutations known to decrease the DNA affinity of Cas9 (ref. 20) that reduces off-target editing events with only a modest decrease in on-target editing activity. Next, we reveal that lipid-mediated delivery of base editor proteins complexed with guide RNA results in even larger specificity enhancements with no apparent reduction in on-target base editing compared to plasmid DNA delivery. Delivery of base editors as ribonucleoproteins (RNPs) typically reduces off-target editing to below measurable levels, even for a notoriously promiscuous guide RNA that targets a highly repetitive genomic DNA sequence, in cultured human and mouse cells. These advances enable us to demonstrate highly specific, DNA-free *in vivo* base editing in mice and zebrafish.

## Results

### Engineering a high-fidelity base editor

**Figure 1:**
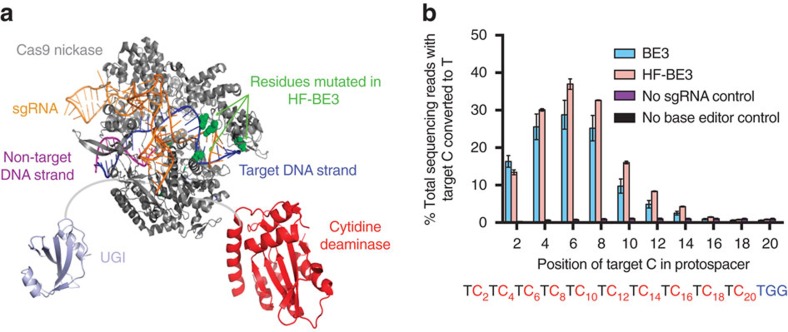
Engineering and *in vitro* characterization of a high-fidelity base editor (HF-BE3). (**a**) Schematic representation of HF-BE3. Point mutations introduced into BE3 to generate HF-BE3 are shown in green. The representation used PDB structures 4UN3 (Cas9), 4ROV (cytidine deaminase) and 1UGI (uracil DNA glycosylase inhibitor). (**b**) *In vitro* deamination of synthetic substrates containing ‘TC' repeat protospacers. Values and error bars reflect the mean and range of two independent replicates performed on different days.

Cas9 nucleases and their associated fusion constructs have been shown to bind and cleave DNA at off-target genomic loci^21^,^22^,^23^,^24^. Joung and co-workers^20^ developed HF-Cas9, a high-fidelity SpCas9 variant containing four point mutations (N497A, R661A, Q695A and Q926A) that were designed to eliminate nonspecific interactions between Cas9 and the phosphate backbone of the DNA target strand (Fig. 1a) consistent with the previous abrogation of nonspecific DNA interactions in TALENs that greatly increased their DNA cleavage specificity^25^. Since base editors operate on the non-target strand within the single-stranded DNA bubble created by Cas9 (ref. 14) we hypothesized that introducing these four point mutations from HF-Cas9 into BE3 to generate ‘HF-BE3' might reduce off-target base editing without altering its base conversion capabilities (Fig. 1a,b).

Plasmids encoding BE3 and HF-BE3 as His_6_-tagged proteins were overexpressed in *Escherichia coli* and purified first by nickel affinity chromatography and then by cation exchange chromatography (Supplementary Fig. 1a,b). Following extensive optimization of expression and purification conditions, BE3 and HF-BE3 protein can be routinely produced at a yield of ∼2 mg l^−1^ of culture media (Supplementary Fig. 1a–c).

We used the purified base editor proteins to compare base-editing efficiency and the width of the editing window of HF-BE3 and BE3 biochemically. We measured *in vitro* C to U conversion efficiencies in a synthetic double-stranded DNA (dsDNA) 79-mer with a protospacer comprised of TC repeats. The target dsDNA (250 nM) was incubated with BE3:sgRNA or HF-BE3:sgRNA (2 μM) for 30 min at 37 °C. After incubation, the edited DNA was amplified using a uracil-tolerant polymerase and sequenced by high-throughput DNA sequencing (HTS). We observed comparable editing efficiencies and activity window widths for HF-BE3 and BE3 *in vitro* (Fig. 1b). These findings indicate that introduction of the high-fidelity mutations into BE3 does not compromise inherent on-target base-editing efficiency or change the width of the editing window of the resulting HF-BE3 protein *in vitro*.

### HF-BE3 enhances editing specificity following transfection

Next, we compared base-editing efficiencies, specificities and editing window widths of BE3 and HF-BE3 in mammalian cells following plasmid DNA transfection. We chose four well-studied endogenous genomic loci (HEK293 site 3, FANCF, EMX1 and VEGFA site 2) to interrogate on- and off-target base editing in mammalian cells^14^,^24^. VEGFA site 2 is highly repetitive, containing 14 Cs out of 20 protospacer nucleotides, and is associated with notoriously high rate of known off-target genome editing^20^,^22^,^24^,^26^. We chose to include this site because it poses a formidable specificity challenge. In contrast with most nuclease-based genome-editing applications, base editing relies on the precise location of the protospacer to place the target nucleotide within the editing window and usually little or no flexibility in the choice of guide RNA is available. Therefore, the development of base editors with enhanced specificities even for highly repetitive, promiscuous sgRNA targets is crucial^3^,^14^.

**Figure 2:**
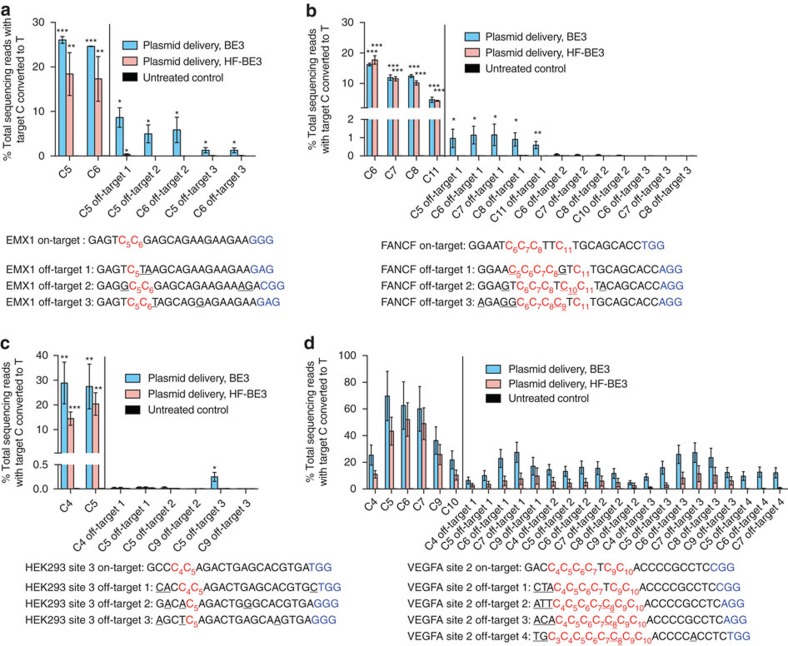
Activity of a high-fidelity base editor (HF-BE3) in human cells. (**a**–**c**) On- and off-target editing associated with plasmid transfection of BE3 and HF-BE3 was assayed using high-throughput sequencing of genomic DNA from HEK293T cells treated with sgRNAs targeting non-repetitive genomic loci EMX1 (**a**), FANCF (**b**) and HEK293 site 3 (**c**). On- and off-target loci associated with each sgRNA are separated by a vertical line. (**d**) On- and off-target editing associated with the highly repetitive sgRNA targeting VEGFA site 2. Values and error bars reflect mean±s.d. of three independent biological replicates performed on different days. For **a**–**c**, stars indicate significant editing based on a comparison between the treated sample and an untreated control. *P≤0.05, **P≤0.01 and ***P≤0.001 (Student's two-tailed *t*-test). For **d**, asterisks are not shown since all treated samples displayed significant editing relative to the control. Individual *P* values are listed in Supplementary Table 1.

We amplified by PCR and analysed by HTS the on-target locus and known off-target loci following plasmid transfection^24^ with each of the four base editor:sgRNA pairs. On-target editing in HEK293T cells for these four endogenous genomic loci was slightly reduced by introduction of the HF mutations; editing averaged 29±5% with BE3, and 21±3% (mean±s.e.m. for *n*=3 biological replicates) for HF-BE3 (Figs 2a–d and 4a).

For each of the three standard, non-repetitive target sites (HEK293 site 3, FANCF and EMX1), we examined the three most frequently modified off-target loci that contain a C within the editing window from the off-target loci previously reported to be modified from treatment with Cas9 and the same guide RNA (Supplementary Table 2)^24^. When cells were transfected with BE3 plasmid, C→T conversion across the nine most frequently modified Cas9 off-target loci for HEK293 site 3, FANCF and EMX1 averaged 1.1±0.3% (Fig. 2a–c; mean±s.d. for *n*=3 biological replicates). Installation of the HF mutations reduced the absolute level of the mean off-target editing by 37-fold to 0.03±0.005%, with only one instance of measureable off-target C→T conversion (Fig. 2a; EMX1 C_5_ at off-target 1).

To characterize HF-BE3 specificity on an extremely challenging site, we compared BE3 and HF-BE3 off-target activity when targeting the highly repetitive VEGFA site 2 locus. BE3 treatment leads to an average of 15±5% editing of cytosines located in the activity windows of the four tested off-target sites associated with this sgRNA (all average values quoted in this paragraph represent mean±s.d. for *n*=3 biological replicates). In contrast, HF-BE3 leads to a threefold reduction in absolute off-target editing (5.0±2.3%) at the same off-target sites (Fig. 2d). When compared to transfection of BE3, HF-BE3 significantly (*P*<0.05, two-tailed Student's *t*-test) reduced off-target editing at 27 of the 57 cytosines located at off-target loci (Supplementary Table 1), while HF-BE3 treatment leads to a significant reduction (*P*<0.05 two-tailed Student's *t*-test) in on-target editing at only 3 of the 16 interrogated on-target cytosine residues.

In addition to considering the differences between absolute editing at off-target loci, we also calculated the on-target:off-target editing specificity ratio by dividing the observed on-target efficiency by the off-target efficiency (Supplementary Fig. 2a,b). This metric takes into account any reduction in on-target editing associated with installation of the HF mutations, and is useful for applications sensitive to both the efficiency and specificity of base editing. Off-target editing by HF-BE3 was below the detection limit of high-throughput sequencing for several off-target loci. For these cases, we assumed a conservative off-target editing efficiency equal to the upper limit of detection (0.025% C→T conversion; see Methods). On the basis of this analysis, the average improvement in specificity ratio upon installation of the HF mutations across all 34 target cytosines we examined was 19-fold, when plasmid delivery of the two constructs was performed. These results collectively establish that for non-repetitive sites (Supplementary Fig. 2a) as well as a highly repetitive site (Supplementary Fig. 2b), HF-BE3, results in a substantially enhanced base-editing specificity with only a modest reduction in on-target editing efficiency compared to BE3.

### RNP delivery of BE3 enables DNA-free base editing

Next, we studied the ability of BE3 in a DNA-free, RNP form to mediate base editing when directly delivered into cultured human cells. We and others recently established that cationic lipid reagents can potently deliver negatively charged proteins or protein:nucleic acid complexes into mammalian cells including ribonucleoprotein (RNP) complexes and that RNP delivery can substantially reduce off-target genome editing^27^,^28^,^29^

**Figure 3:**
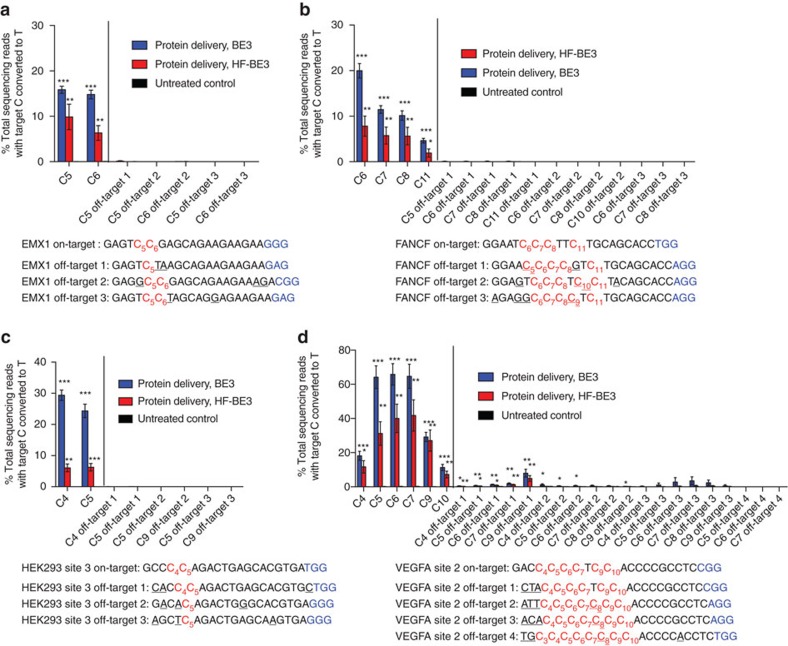
Protein delivery of base editors into cultured human cells. (**a**–**d**) On- and off-target editing associated with RNP delivery of base editors complexed with sgRNAs targeting EMX1 (**a**), FANCF (**b**), HEK293 site 3 (**c**) and VEGFA site 2 (**d**). Off-target base editing was undetectable at all of the sequenced loci for non-repetitive sgRNAs. Values and error bars reflect mean±s.d. of three independent biological replicates performed on different days. Stars indicate significant editing based on a comparison between the treated sample and an untreated control. *P≤0.05, **P≤0.01 and ***P≤0.001 (Student's two-tailed *t*-test).

**Figure 4:**
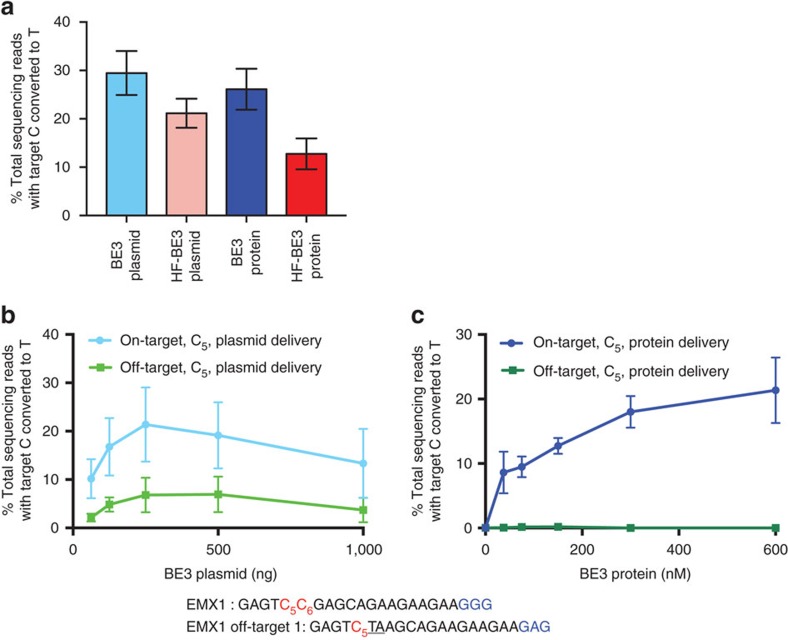
Effect of dosage of BE3 protein or plasmid on the efficiency of on-target and off-target base editing in cultured human cells. (**a**) On-target editing efficiency at each of the four genomic loci was averaged across all edited cytosines in the activity window for each sgRNA. Values and error bars reflect mean±s.e.m. of three independent biological replicates performed on different days. (**b**,**c**) On- and off-target editing at the EMX1 site arising from BE3 plasmid titration (**b**) or BE3 protein titration (**c**) in HEK293T cells. Values and error bars reflect mean±s.d. of three independent biological replicates performed on different days.

We combined the commercially available cationic lipid Lipofectamine 2000 with either purified BE3 protein or HF-BE3 protein after pre-complexation with a guide RNA targeting the EMX1, HEK293 site 3, FANCF or VEGFA site 2 locus, and incubated the resulting lipid:RNP complexes with HEK293T cells. After 72 h, we harvested genomic DNA and analysed on-target and off-target base editing by high-throughput DNA sequencing. As with all Cas9-based technologies, we observed substantial variations in editing efficiency at different genomic loci (Figs 2 and 3). To display trends associated with on-target editing efficiency between different treatments, we calculated the mean on-target base-editing efficiencies at the four tested loci (Fig. 4a). Protein delivery of BE3 (200 nM) leads to on-target editing efficiencies comparable to those observed with plasmid transfection (26±4% versus 29±5%, respectively; mean±s.e.m. for *n*=3 biological replicates; Fig. 4a).

In contrast, protein delivery of HF-BE3 reduced on-target editing compared to protein delivery of BE3 at the four genomic loci studied (average editing efficiency of 13±3% versus 26±4%, respectively; mean±s.e.m. for *n*=3 biological replicates; Fig. 4a). Since HF-BE3 and BE3 have comparable editing efficiencies in a test tube (Fig. 1b) and editing is only slightly reduced when HF-BE3 is expressed from plasmids in HEK293T cells (Fig. 2a–d), it is tempting to speculate that the decreased efficiency of editing from HF-BE3 protein delivery may be a result of decreased HF-BE3 stability in mammalian cells. Lower stability could be offset by continual expression from a plasmid, but not following one-time protein delivery. This observation is consistent with a recent report of reduced on-target indel formation with purified HF-Cas9 compared to purified Cas9 when nucleofected into CD34^+^ haematopoietic stem and progenitor cells^30^. While this work was in review, Kim *et al*.^31^ demonstrated RNP delivery of BE3 into mouse embryos using electroporation. To the best of our knowledge, our approach is the first DNA-free technique capable of generating precise changes to individual nucleotides in mammalian cells without electroporation, which has limited *in vivo* therapeutic relevance.

### RNP delivery of base editors greatly enhances specificity

Importantly, while RNP delivery of BE3 and HF-BE3 led to substantial on-target base editing, we observed no instances of measurable base editing (<0.025%) at any of the nine tested off-target loci associated with EMX1, FANCF and HEK293 site 3 (Fig. 3a–c). In contrast, plasmid delivery of BE3 leads to an average of 1.1±0.3% (mean±s.d. for *n*=3 biological replicates) off-target editing across all sequenced cytosines within the base-editing activity window, and detectable off-target editing at 11 of the 16 off-target cytosines located at these nine off-target loci (Fig. 2a–d). At off-target loci of the three non-repetitive loci tested, BE3 protein delivery leads to a 26-fold higher average specificity ratio than that of plasmid delivery (Supplementary Fig. 2a). These results reveal that RNP delivery of base editors markedly increases the DNA specificity of base editing.

Protein delivery of either BE3 or HF-BE3 also resulted in greatly improved base-editing specificity at the highly promiscuous VEGFA site 2 locus compared to plasmid delivery of either BE3 or HF-BE3 (compare Figs 2 and 3; see Supplementary Table 1). Absolute frequencies of base editing at the off-target loci associated with this site were reduced upon protein delivery at least 10-fold for both BE3 (plasmid delivery: 15±4% off-target editing; protein delivery: 1.3±0.4% off-target editing; all values in this paragraph represent mean±s.d. for *n*=3 biological replicates) and HF-BE3 (plasmid delivery: 5±2% off-target editing; protein delivery: 0.5±0.1% off-target editing). Across all four studied loci, base-editing specificity ratios for on-target:off-target editing increased an average of 66-fold for protein delivery of BE3 compared with plasmid delivery of BE3 (Supplementary Fig. 2). Collectively, these results reveal that for both repetitive and non-repetitive target sites, RNP versus DNA delivery is a stronger determinant of base-editing specificity than the presence or absence of the high-fidelity Cas9 mutations.

Neither introduction of the HF mutations nor delivery method substantially altered the low indel rates associated with base editing. Indel frequencies at all on-target loci across all treatment conditions in this study remained low (typically ≤5%; Supplementary Fig. 3a), and the editing:indel ratio remained higher in all cases tested (typically≥10-fold; Supplementary Fig. 3b) than in previous studies using optimized HDR protocols^30^,^32^,^33^. For non-repetitive sgRNAs, very few indels were observed at off-target loci (Supplementary Fig. 3c), although we note that plasmid delivery of BE3 generated up to 5% indels for off-target loci associated with VEGFA site 2 (Supplementary Fig. 3c).

Taken together, these results establish that protein delivery of base editors maintains on-target base-editing efficiency and greatly enhances editing specificity relative to delivery of plasmid DNA.

### RNP delivery decouples on- and off-target editing

Given the striking enhancement of base-editing specificity associated with protein delivery of BE3, we investigated whether this improvement was a result of a reduction in the total quantity of active genome-editing agent delivered into the cell. Using the sgRNA targeting EMX1, we performed a dose response study for plasmid (Fig. 4b) and protein delivery (Fig. 4c). To maximize transfection efficiency between treatment conditions, the volume of Lipofectamine 2000 was 1.5 μl for all tests, and the base editor protein:sgRNA molar ratio was maintained at 1:1.1 for protein delivery. For plasmid delivery, we used a mass ratio of sgRNA plasmid:BE3 plasmid of 1:3 (molar ratio ∼1:1) and 1.5 μl of Lipofectamine 2000. We observed off-target base editing under all conditions tested for plasmid delivery (Fig. 4b), but virtually no off-target editing under all protein delivery conditions tested (Fig. 4c).

We performed linear regression analysis to assess the relationship between on- and off-target editing for plasmid and protein delivery. For plasmid delivery, off-target editing was closely associated with on-target editing rates (*R*^2^=0.95, *P*=0.0012 for non-zero slope, F-test), whereas there was no significant association between off-target and on-target editing using protein delivery (*R*^2^=0.078, *P*=0.59 for non-zero slope, F-test).

These data indicate that protein delivery of base editors offers an inherent specificity advantage that is independent of dosage. Together with our previous observations^29^,^34^, these findings support a model in which the higher DNA specificity of base editing from protein delivery compared to DNA delivery arises from the ability of protein delivery to avoid extended exposure of the genome to base editors, thereby minimizing the opportunity of base editors to process off-target loci after on-target loci have already been modified.

### DNA-free base editing in zebrafish and mice

**Figure 5:**
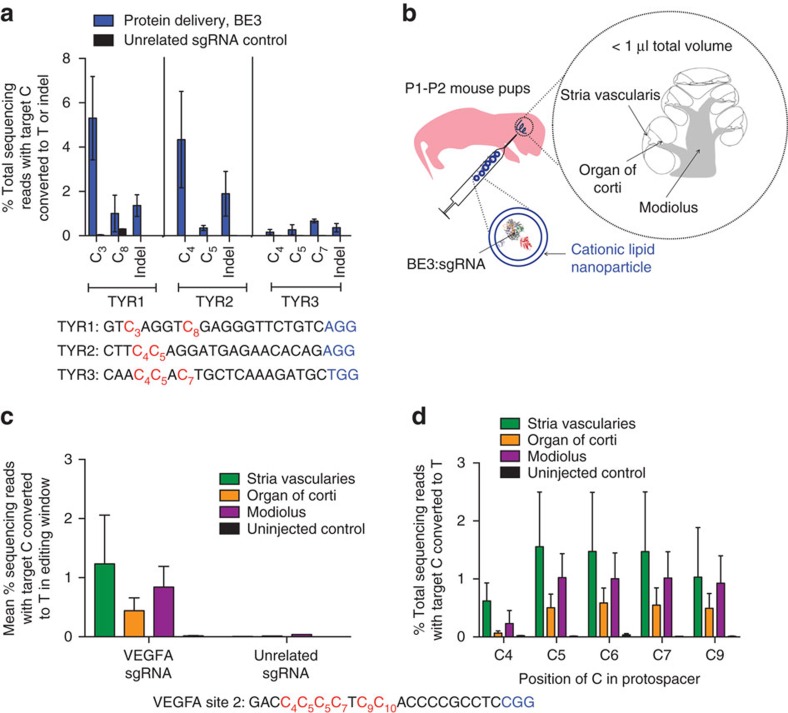
DNA-free *in vivo*base editing in zebrafish embryos and in the inner ear of live mice using RNP delivery of BE3. (**a**) On-target genome editing in zebrafish harvested 4 days after injection of BE3 complexed with indicated sgRNA. Values and error bars reflect mean±s.d. of three injected and three control zebrafish. Controls were injected with BE3 complexed with an unrelated sgRNA. (**b**) Schematic showing *in vivo* injection of BE3:sgRNA complexes encapsulated into cationic lipid nanoparticles. (**c**) Base editing of cytosine residues in the base editor window at the VEGFA site 2 genomic locus. (**d**) On-target editing at each cytosine in the base-editing window of the VEGFA site 2 target locus. (**c**,**d**) Values and error bars reflect mean±s.e.m. of three mice injected with sgRNA targeting VEGFA site 2, three uninjected mice and one mouse injected with unrelated sgRNA.

The above observations suggested the promise of protein delivery of BE3 to maintain on-target base editing while eliminating detectable off-target base editing. We therefore tested whether protein delivery of BE3 could be used to generate specific point mutations in zebrafish by injecting BE3:sgRNA complexes targeting the *tyrosinase* locus into fertilized zebrafish embryos. We harvested genomic DNA from the resultant zebrafish larvae 4 days post injection and measured base editing and indel frequencies by high-throughput sequencing (Fig. 5a). Two of the three BE3:sgRNA complexes tested induced substantial point mutations *in vivo* (TYR1: C_3_→T_3_ 5.3±1.8%, TYR2: C_4_→T_4_ 4.3±2.1%; mean±s.d. of *n*=3 injected embryos; Fig. 5a). Sequences of zebrafish loci are listed in Supplementary Table 5.

Finally, we applied these developments to achieve DNA-free, high-specificity base editing in mice. To maximize the likelihood of observing on- and off-target base editing *in vivo*, we used the highly repetitive sgRNA targeting VEGFA site 2; conveniently, the murine and human genomes are identical at this target site.

Using cultured murine NIH/3T3 cells, we confirmed that BE3 protein delivery yielded efficient on-target base editing at this locus 34±11% (Supplementary Fig. 4a; all editing percentages in this paragraph represent mean±s.d. for *n*=3 biological replicates). We used the Cutting Frequency Determinant (CFD) algorithm^29^,^34^ to predict off-target loci in the mouse genome associated with the VEGFA site 2 sgRNA (Supplementary Table 3). Using cultured NIH/3T3 cells, we confirmed that two of the top four predicted off-target loci are indeed modified by plasmid delivery of BE3 in cultured murine cells (CFD off-target locus 1; mean editing 9±5% and CFD off target locus 4; mean editing 3±2%; Supplementary Fig. 4b–e). Consistent with our results from human cells, protein delivery of BE3 reduced off-target editing to levels similar to that of negative controls (Supplementary Fig. 4c,e). The mean base-editing specificity ratio for CFD off-target loci 1 and 4 increased from 28±13 for plasmid delivery of BE3 to ≥780±300 for protein delivery of BE3 (values represent mean±s.e.m.; *n*=3 biological replicates).

To establish DNA-free base editing in mice, we combined BE3:sgRNA complexes with Lipofectamine 2000 (Fig. 5b) and performed intracochlear injections into mouse pups at P1–P2. Injected cochlear tissues were harvested 3–4 days post injection and microdissected into five to seven samples per cochlear region. Control cochlea from uninjected mice were harvested simultaneously. Genomic DNA was extracted from the harvested tissue, amplified by quantitative PCR (qPCR) to late-exponential phase, and subjected to high-throughput DNA sequencing to measure C→T conversion. Although it is impossible to quantitate base-editing efficiency among treated cells because it is not possible to retrieve DNA exclusively from cells exposed to base editor protein, we observed unambiguous base editing from tissue in three regions of the cochlea: the basal end of the organ of Corti, the stria vascularis and the modiolus (Fig. 5c,d). We detected no significant indel formation in treated tissue samples (<0.1% indels; Supplementary Fig. 5b).

The percentage of cochlear cells containing target C→T conversion (Fig. 5c) was significantly lower than that observed in treated NIH/3T3 cells in culture (Supplementary Fig. 4a), consistent with the highly localized nature of lipid-based protein delivery and our inability to isolate DNA exclusively from cells exposed to base editor. Nonetheless, local delivery offers key advantages for accessible applications, including control over which cell types are edited, and ease of preparation and administration.

Finally, we analysed off-target editing following intracochlear injection of BE3:sgRNA:lipid complexes. Analysis of all four predicted off-target loci, including the confirmed off-target sites CFD locus 1 and CFD locus 4 in genomic DNA from the cochlear tissue of mice injected with the BE3:VEGFA site 2 sgRNA:lipid complex revealed no detectable C→T conversion or indel formation above that observed in untreated control samples for any of the off-target loci tested (Supplementary Fig. 5a).

Together, these *in vivo* base-editing results establish a virus-free, DNA-free strategy for the precise conversion of individual nucleotides in the genomic DNA of animals with high DNA sequence specificity.

## Discussion

The strategies developed and implemented in this study expand the utility and applicability of base editing by removing or reducing off-target base editing and establishing a DNA-free delivery method that supports *in vivo* base editing. Protein delivery improves base-editing specificity in human and murine cells compared with plasmid delivery of the same constructs (Figs 2 and 3 and Supplementary Fig. 4), and enables specific base editing in zebrafish and in the mouse cochlea (Fig. 5).

We generated a high-fidelity base editor by installing into BE3 mutations known to enhance the DNA specificity of Cas9 (ref. 20). The installation of these mutations into Cas9 was reported to result in undetectable indel formation at off-target loci associated with non-repetitive sgRNAs, including the EMX1 locus interrogated here (Fig. 2a)^20^. The specificity enhancements we observed in HF-BE3, while substantial, were more modest; HF-BE3 exhibited detectable off-target base editing at both repetitive and non-repetitive loci when delivered as plasmid DNA into mammalian cells (Fig. 2a,d and Supplementary Fig. 4c,e). It is tempting to speculate that this specificity enhancement difference may arise from the fact that base editing, unlike Cas9-mediated indel formation, does not require DNA cleavage but only necessitates DNA-binding and R-loop formation^14^, and some of the enhanced specificity of HF-Cas9 may arise from impaired DNA cleavage at already-bound off-target loci.

In a second attempt to reduce off-target base editing, we demonstrated that RNP delivery of base editors leads to decoupling of on- and off-target editing (Fig. 4b,c). RNP delivery ablated off-target editing at non-repetitive sites while maintaining on-target editing comparable to plasmid delivery (Figs 3a–c and 4a), and greatly reduced off-target editing even at the highly repetitive VEGFA site 2 (Fig. 3d). RNP delivery of base editors may be especially useful for *in vivo* editing applications in which cellular dosage is typically difficult to control or characterize.

We and others previously used RNP delivery of Cas9 coupled with the delivery of a donor DNA template to perform HDR-based genome editing in mammalian cells. These approaches, however, remain limited by low efficiency, cell-state dependence and indel formation efficiencies typically exceeding those of desired HDR outcomes, especially for point mutation correction^29^,^30^,^32^,^35^. DNA-free base editing, in contrast, generates a substantial excess of edited product relative to stochastic indels both *in vivo* and in cells (Fig. 5a and Supplementary Fig. 5a,b). To the best of our knowledge, RNP delivery of base editors represents the first strategy for generating specific and precise modifications to genomic DNA without requiring exogenous DNA.

## Methods

### Cloning of plasmids

The plasmids in this study were generated by USER cloning. Phusion U Hot Start polymerase (Thermo Fisher) was used to install point mutations and construct protein expression plasmids from previously reported constructs^36^. Protein sequences are listed in the Supplementary Information, and plasmids for expression of BE3 and HF-BE3 are available from Addgene.

### Expression and purification of BE3 and HF-BE3

BL21 Star (DE3)-competent *E. coli* cells were transformed with plasmids encoding the bacterial codon-optimized base editors with a His_6_ N-terminal purification tag. A single colony was grown overnight in Luria-Bertani broth containing 50 μg ml^−1^ kanamycin at 37 °C. The cells were diluted 1:200 into 2 l of the same media and grown at 37 °C until OD_600_=0.70–0.75. The cultures were incubated on ice for 60 min and protein expression was induced with 0.5 mM isopropyl-β-D-1-thiogalactopyranoside (GoldBio). Expression was sustained for 14–16 h with shaking at 18 °C. The subsequent purification steps were carried out at 4 °C. Cells were collected by centrifugation at 6,000*g* for 20 min and resuspended in cell collection buffer (100 mM tris(hydroxymethyl)-aminomethane (Tris)-HCl, pH 8.0, 1 M NaCl, 20% glycerol, 5 mM tris(2-carboxyethyl)phosphine (TCEP; GoldBio), 0.4 mM phenylmethane sulfonyl fluoride (Sigma-Aldrich) and 1 complete, EDTA-free protease inhibitor pellet (Roche) per 50 ml buffer used). Cells were lysed by sonication (6 min total, 3 s on, 3 s off) and the lysate cleared by centrifugation at 25,000*g* (20 min).

The cleared lysate was incubated with His-Pur nickel nitriloacetic acid (nickel-NTA) resin (1 ml resin per litre of culture, Thermo Fisher) with rotation at 4 °C for 60–90 min. The resin was washed with 20 column volumes of cell collection buffer before bound protein was eluted with elution buffer ((100 mM tris(hydroxymethyl)-aminomethane (Tris)-HCl, pH 8.0, 0.5 M NaCl, 20% glycerol, 5 mM TCEP (GoldBio), 200 mM imidazole). The resulting protein fraction was further purified on a 5 ml Hi-Trap HP SP (GE Healthcare) cation exchange column using an Akta Pure FPLC. Protein-containing fractions were concentrated using a column with a 100,000 kDa cutoff (Millipore) centrifuged at 3,000*g*, and the concentrated solution was sterile-filtered through a 22-μm polyvinylidene difluoride membrane (Millipore).

After sterile filtration, proteins were quantified with Reducing Agent Compatible Bicinchoninic acid assay (Pierce Biotechnology), snap-frozen in liquid nitrogen and stored in aliquots at −80 °C. Sequences of expressed proteins are listed in Supplementary Note 2.

### *In vitro* transcription of sgRNA

Linear DNA fragments containing the T7 RNA polymerase promoter sequence upstream of the desired 20 bp sgRNA protospacer and the sgRNA backbone were generated by PCR (Q5 Hot Start MasterMix, New England Biolabs) using primers as listed in the Supplementary Note 3 and concentrated on minelute columns (Qiagen). sgRNA was transcribed with the HiScribe T7 High Yield RNA Synthesis Kit (New England Biolabs) at 37 °C for 14–16 h with 1 μg of linear template per 20 μl reaction. sgRNA was purified using the MEGAClear Transcription Clean Up Kit (Thermo Fisher), according to the manufacturer's instructions. Purified sgRNAs were stored in aliquots at −80 °C.

### *In vitro* deamination assays

Sequences of DNA oligonucleotides used as templates for the *in vitro* deamination assay are shown in Supplementary Note 3. All oligonucleotides were purchased from IDT. Single-stranded oligonucleotides synthesized with complementary sequences were combined (5 μl of a 100 μM solution) in Tris buffer pH 8.0 and annealed by heating to 95 °C for 5 min, followed by a gradual cooling to 37 °C at a rate of 0.1 °C s^−1^ to generate 79 base pair (bp) dsDNA substrates. Freshly thawed base editor proteins (2 μM final concentration in a 10 μl reaction volume) were complexed with the indicated sgRNA (2.2 μM final concentration) in reaction buffer (20 mM HEPES pH 7.5, 150 mM KCl, 0.5 mM dithiothreitol (DTT), 0.1 mM EDTA, 10 mM MgCl_2_)^37^ for 5 min at room temperature. Annealed dsDNA substrates were then added to a final concentration of 250 nM. The reaction proceeded for 30 min at 37 °C before protein denaturation was performed by heating for 5 min at 99 °C. Addition of PB buffer (Qiagen, 100 μl) and isopropanol (25 μl)-ensured protein was dissociated from the substrate DNA. DNA was purified with Minelute columns (Qiagen), and the resulting products amplified to the top of the linear range with 15 cycles of qPCR (12 ng input DNA, 50 μl reaction volume) using a U-tolerant polymerase (Phusion U Hot Start, Thermo Fisher) and primers as listed in the Supplementary Information. Amplified DNA was purified using RapidTip2 (Diffinity Genomics) and barcoded with a second round of PCR (eight cycles, 5 ng input) before being prepared for sequencing on an Illumina MiSeq as described below.

### Purification and sequencing of genomic DNA

Genomic DNA was isolated using the Agencourt DNAdvance Genomic DNA Isolation Kit (Beckman Coulter) according to the manufacturer's instructions. For the first PCR, DNA was amplified to the top of the linear range using Q5 Hot Start DNA Polymerase (NEB), according to the manufacturer's instructions but with the addition of 3% dimethylsulphoxide and SYBR Gold Nucleic Acid Stain (Thermo Fisher). For all amplicons, the PCR protocol used was an initial heating step of 2 min at 98 °C followed by an optimized number of amplification cycles (12 s at 98 °C, 25 s at 61 °C, 30 s at 72 °C). For zebrafish and for transfected cell samples 30 ng of input DNA was used in a 50 μl reaction, and for cochlear samples 20 ng was used in a 25 μl reaction. qPCR was performed to determine the optimal cycle number for each amplicon. Amplified DNA was purified using RapidTip2 (Diffinity Genomics) and barcoded with a further PCR (eight cycles, 5 ng input). The unique forward and reverse primers used in the first-round PCR contained a constant region 5′ to the annealing region, (forward: 5′-ACACTCTTTCCCTACACGACGCTCTTCCGATCTNNNN-3′, reverse: 5′-TGGAGTTCAGACGTGTGCTCTTCCGATCT-3′), which facilitated the binding of barcoding primers to amplified DNA for a second-round PCR. In brief, an annealing temperature of 60 °C was used and cycle numbers were 30 (EMX1), 28 (FANCF) and 28 (HEK site 3).

The second-round PCR used primers with three regions: a 5′ constant region allowing the amplicon to bind to the Illumina flow cell (italicized), an eight-base barcoding region (X) and a 3′ constant region allowing the barcoding primer to bind to the first-round PCR amplicon (in bold). Examples of primer sequences are:

forward: 5′-*AATGATACGGCGACCACCGAGATCTACAC*XXXXXXXX**ACACTCTTTCCCTACACGAC-**3′

reverse: 5′-CAAGCAGAAGACGGCATACGAGATXXXXXXX**GTGACTGGAGTTCAGACGTGTGCTCTTC-**3′.

Sequencing adapters and dual-barcoding sequences are based on the TruSeq Indexing Adapters (Illumina). Barcoded samples were pooled and purified by gel extraction (Qiagen), and then purified using Ampure beads (Beckman Coulter) before quantification using the Qubit dsDNA HS Kit (Thermo Fisher) and qPCR (KAPA BioSystems) according to the manufacturer's instructions. Sequencing of pooled samples was performed using a single-end read from 180 to 250 bases (depending on the amplicon size) on the MiSeq (Illumina) according to the manufacturer's instructions.

Sequences of oligonucleotides used for PCR amplification are shown in Supplementary Note 3. All oligonucleotides were obtained from IDT. The optimized number of PCR cycles for each amplicon in this study are as follows: VEGFA site 2 human genomic DNA (annealing temperature was 61 °C for 25 s for all extension steps): on-target: 29 cycles, off-target #1: 32 cycles, off-target #2:28 cycles, off-target #3:27 cycles, off-target #4:27 cycles, VEGFA site 2 murine genomic DNA: on-target: 31 cycles, off-targets #1, #2, #3 and #4:31 cycles. HEK293 site 3: off-targets #1:29 cycles, off-target #2:28 cycles, off-target #3:28 cycles. FANCF off-target #1:29 cycles, off-target #2:28 cycles, off-target 3:28 cycles. EMX1 off-targets #1, #2 and #3:28 cycles. TYR1, TYR2 and TYR3 sgRNAs for amplification of zebrafish DNA: 32 cycles. Optimized protocols for the on-target amplification of the EMX1, FANCF and HEK293 site 3 loci were followed as previously described^14^.

### Analysis and alignment of genomic DNA sequencing reads

Sequencing reads were analysed as previously described^14^. In brief, sequencing reads were demultiplexed using MiSeq Reporter (Illumina), and individual FASTQ files were analysed with a previously reported custom Matlab script^14^. Reads were aligned to the reference sequence using the Smith-Waterman algorithm. Base calls with *Q*-scores below 30 were replaced with a placeholder nucleotide (N). This quality threshold results in nucleotide frequencies with an expected error rate of 1 in 1,000. Indel frequencies were quantified with a previously published custom Matlab script, which counts indels occurring in a 30-base window around the nCas9 cleavage site and are a minimum of two-base insertions or deletions^14^. Indels were defined as detectable if there was a significant difference (Student's two-tailed *t*-test, *P*<0.05) between indel formation in the treated sample and untreated control.

For one of the sequenced amplicons, CFD off-target #3, associated with VEGFA site 2 sgRNA in the murine genome, it was not possible to accurately measure indel formation. The protospacer at this locus is directly preceded by 12 guanine bases, which makes PCR and high-throughput sequencing of this site prone to random insertion or deletions; deletion rates as high as 20% of sequencing reads were observed in multiple independent untreated control samples. Since no significant base editing was detected at this off-target locus under any treatment conditions (Fig. 5 and Supplementary Fig. 4d), we suspect that indel formation is also negligible at this locus.

A phred.II Q30 score corresponds to an estimated 99.9% accuracy in base calling^38^. A 0.1% probability of incorrect base calling at a given position corresponds to a lower limit for base calling of 0.1/4=0.025% if we assume base call errors are randomly distributed across the four bases. C→T editing percentages that fell beneath this threshold were classified as undetectable. Spontaneous deamination^39^ or polymerase error during PCR can also introduce artefactual C→T edits. In order to distinguish base editor-induced C→T editing from artefactual C→T editing rates, we sequenced untreated control cells for each amplicon and calculated whether the C→T editing under a particular condition was statistically significant using the Student's two-tailed *t*-test with *P*<0.05 as the threshold. Off-target sites with statistically significant editing rates >0.025% were considered measureable. The number of aligned and quality-filtered reads for each sample has been included in Supplementary Table 5.

### Statistical analyses of genomic DNA sequence alignments

Unless otherwise noted, the mean values cited throughout the main text are representative of *n*≥3 independent biological replicates and the mean±s.d. has been stated.

The statistical analysis of the high-throughput sequencing data displayed in Figs 2 and 3 was performed by comparing on- and off-target editing percentages in treated samples to any editing measured in a negative control sample (untreated). The Student's two-tailed *t*-test was used, and individual *P* values are shown in Supplementary Table 1. *P≤0.05, **P≤0.01 and ***P≤0.001. When editing was below the detection limit (0.025%), significance was not calculated; all untreated control samples showed undetectable editing.

For Fig. 4a, the mean on-target base editing was calculated by averaging editing of cytosines in the base-editing activity window (C_4_–C_8_ for HEK293 site 3 and EMX1, C_4_–C_9_ for FANCF and VEGFA site 2).

To account for sgRNA-dependent differences in base-editing activity, the base editing:indel ratio was calculated (Supplementary Fig. 3b). This ratio was generated by dividing the percentage of HTS reads with a C→T conversion (averaged across the base-editing window for each site) by the percentage of HTS reads containing an indel. As described above, if the off-target editing for a particular locus was below the limit of detection, we conservatively assumed the estimated upper bound of our detection method (0.025%) for the purpose of calculating specificity ratios.

### Data analysis of *in vitro* edited DNA

Sequencing reads were automatically demultiplexed using MiSeq Reporter (Illumina.). Quality-filtering was performed using the online package usegalaxy.org^40^. Individual bases with an Illumina quality score less than or equal to 30 were converted to the placeholder nucleotide ‘N' using FASTQ Groomer followed by FASTA Masker^41^. The resulting quality-filtered FASTQ files were subsequently analysed with a custom python script provided in Supplementary Note 1. Sequencing reads were scanned for exact matches to two 14-base sequences that flank both sides of the target DNA sequence. If no exact matches were found, the read was excluded from analysis. If both 14-base sequences were located and the length of the sequence between them was equal to the expected protospacer length (20 bases), the protospacer sequence found between the flanking regions was saved and the bases called by high-throughput sequencing at each site within the protospacer were tallied.

### Cell culture

Both HEK293T (American Type Culture Collection (ATCC) CRL-3216) and NIH/3T3 (ATCC CRL-1658) were maintained in DMEM plus GlutaMax (Thermo Fisher) supplemented with 10% (v/v) fetal bovine serum, at 37 °C with 5% CO_2._ Cells were obtained from ATCC and were authenticated and verified to be free of mycoplasma by ATCC upon purchase.

### Plasmid transfection of base editors into HEK293T cells

HEK293T cells were seeded on 48-well collagen-coated BioCoat plates (Corning) in an antibiotic-free medium and transfected at ∼70% confluency. Unless otherwise noted, 750 ng of BE and 250 ng of sgRNA expression plasmids were transfected using 1.5 μl of Lipofectamine 2000 (Thermo Fisher) per well according to the manufacturer's protocol.

### Protein transfection of base editors into HEK293T cells

HEK293T cells were seeded on 48-well collagen-coated BioCoat plates (Corning) in 250 μl an antibiotic-free medium and transfected at ∼70% confluency. Base editor protein was incubated with 1.1 × molar excess of the necessary sgRNA at room temperature for 5 min. The complex was then incubated with 1.5 μl Lipofectamine 2000 (Thermo Fisher) and transfected according to the manufacturer's protocol for plasmid delivery. Unless otherwise noted, BE protein was added to a final concentration of 200 nM (based on a total well volume of 275 μl).

### Plasmid transfection of base editors into NIH/3T3 cells

NIH/3T3 cells were seeded on 48-well collagen-coated BioCoat plates (Corning) in an antibiotic-free DMEM medium and transfected at ∼75% confluency. Unless otherwise noted, 600 ng of BE and 200 ng of sgRNA expression plasmids were transfected using 1.4 μl of Lipofectamine 3000 with 1 μl of P3000 reagent (Thermo Fisher) per well according to the manufacturer's protocol.

### Protein transfection of base editors into NIH/3T3 cells

NIH/3T3 cells were seeded on 48-well collagen-coated BioCoat plates (Corning) in an antibiotic-free DMEM medium and transfected at ∼75% confluency. Base editor proteins were incubated with 1.1-fold molar excess of the indicated sgRNA at 25 °C for 5 min. The complex was then incubated with 1.4 μl Lipofectamine 3000 (Thermo Fisher) and transfected according to the manufacturer's protocol for plasmid delivery. P3000 reagent was not used because its addition leads to protein precipitation and a reduction in base-editing efficiency. Unless otherwise noted, BE protein was added to a final concentration of 400 nM (based on a total well volume of 275 μl).

### Intracochlear delivery of BE3 protein:guide RNA

All animal experiments were approved by the Institutional Animal Care and the Use Committee of the Massachusetts Eye and Ear Infirmary. Intracochlear delivery was performed in P1–P2 mice of a mixed genetic background as described previously^42^. Briefly, mice were anaesthetized by lowering the body temperature before the surgical procedure. A postauricular incision was made near the right ear, and the bulla was lifted to expose the cochlea. BE3 protein (57.7 μM stock concentration) was pre-complexed with the sgRNA (100 μM stock concentration) in a 1:1.1 molar ratio and then mixed with Lipofectamine 2000 (Thermo Fisher) in a 1:1 volumetric ratio. The resulting solution (1.2–1.5 μl) was injected with a glass pipette (end diameter, 5 μm) through the cochlear capsule into scala media at the cochlear basal turn that attached to a nanolitre micropump (WPI, UMP3+Micro4+NanoFil) at the rate of 250 nl min^−1^. After injection, the incision was closed and the mice were brought onto a heating pad to recover. After 3–4 days, the cochlea of mouse was dissected into the organ of Corti, stria vascularis and modiolus. Each tissue was further microdissected into between five and seven separate pieces, and DNA extraction was performed separately for each sample, followed by high-throughput sequencing as described above. The data presented in Fig. 5 and Supplementary Figure 5 show sequencing data resulting from extraction of one microdissected sample for each cochlear region.

### Microinjection of base editor RNP into zebrafish embryos

Zebrafish (Tuebingen strain) were maintained under standard conditions in compliance with the internal regulatory review at Boston Children's Hospital. One-cell stage zebrafish embryos were injected with ∼2 nl of BE3 protein pre-complexed with the appropriate sgRNA or an unrelated sgRNA control in a 1:1 molar ratio (4.5 μM final concentration). Four days post fertilization, DNA was extracted from larvae as previously described^43^; in brief, each larva was resuspended in 50 μM NaOH for 30 min at 95 °C and the resulting solution was neutralized with Tris-HCl. Genomic DNA was quantified, amplified by PCR and sequenced as described above.

### Protein gel analyses

All protein gels shown were precast 4–12% polyacrylamide Bis-Tris Plus (Thermo Fisher). They were run in MOPS buffer (Thermo Fisher) at 180 V for 50 min. Samples were prepared for loading by heating to 99 °C in 100 mM DTT and 1 × lithium dodecyl sulfate Sample Buffer for denaturation (Thermo Fisher) for 10 min. Gels were stained using Instant Blue Protein Stain (Expedion) according to the manufacturer's instructions.

For cell lysate analysis, 2 ml of post-induction overnight culture was pelleted at 15,000*g* before lysis in 100 μl B-PER (Thermo Fisher) according to the manufacturer's instructions.

### Data availability

High-throughput sequencing data that support the findings of this study have been deposited in the NCBI Sequence Read Archive database under Accession Number SRP097884. Plasmids encoding HF-BE3 and BE3 for protein expression, as well as HF-BE3 for mammalian expression, are available from Addgene with Accession IDs 87439 (pCMV-HF-BE3), 87438 (pET42b-HF-BE3), 87437 (pET42b-BE3).

## Additional information

**How to cite this article:** Rees, H. A. *et al*. Improving the DNA specificity and applicability of base editing through protein engineering and protein delivery. *Nat. Commun.* **8,** 15790 doi: 10.1038/ncomms15790 (2017).

**Publisher's note**: Springer Nature remains neutral with regard to jurisdictional claims in published maps and institutional affiliations.

## Supplementary Material

Supplementary Information Supplementary Figures, Supplementary Notes, Supplementary Tables, and Supplementary References
