# Supplementary material for: Improving the DNA specificity and applicability of base editing through protein engineering and protein delivery
Source: Nat Commun. 2017 Jun 6;8:15790. doi: 10.1038/ncomms15790 (PMC5467206; doi:10.1038/ncomms15790)
Supplement: Supplementary Information — Supplementary Figures, Supplementary Notes, Supplementary Tables, and Supplementary References [file ncomms15790-s1.pdf]

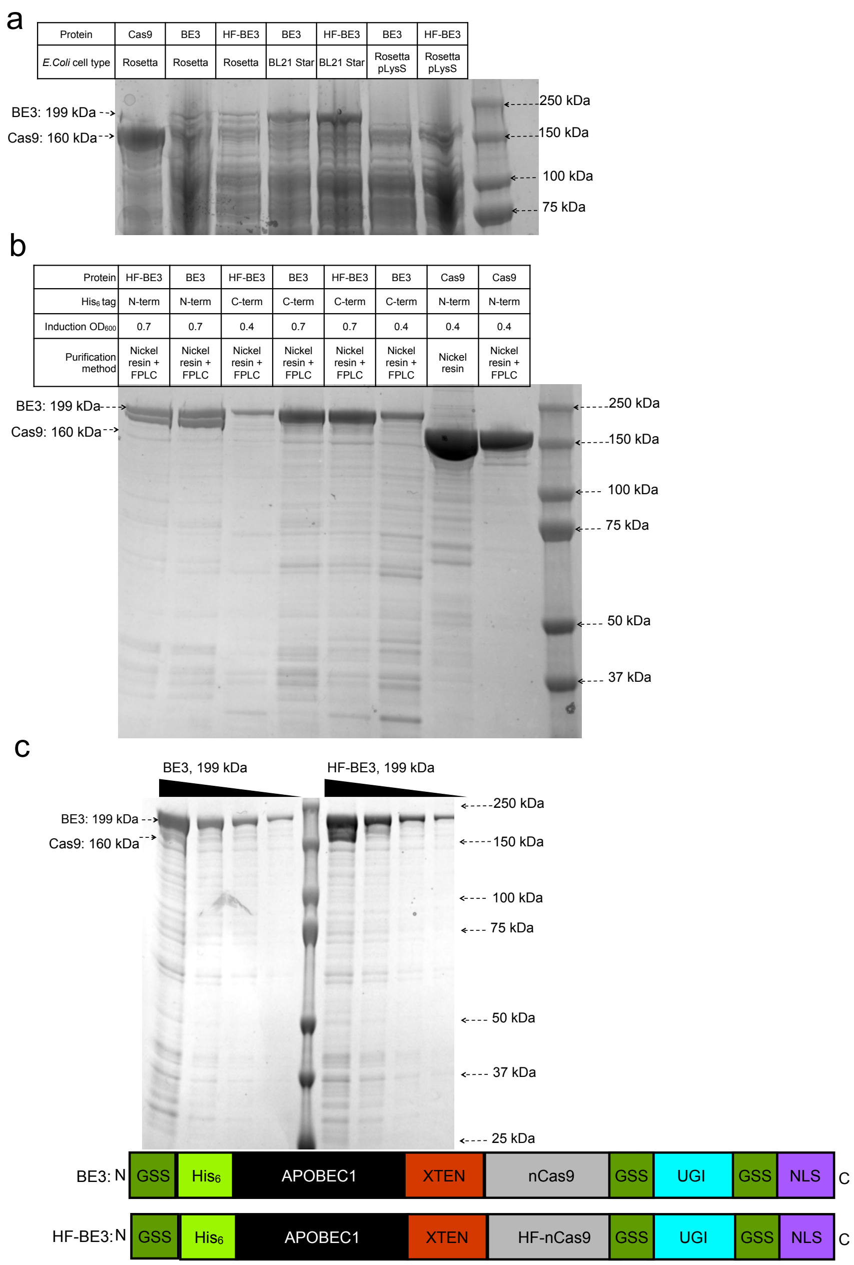

Supplementary Figure 1: **Purification of base editor proteins**. (a): Selection of optimal *E. coli* strain for base editor expression. After IPTG-induced protein expression for 16 h at 18 °C, crude cell lysate was analyzed for protein content. BL21 Star (DE3) (Thermo Fisher) cells showed the most promising post-expression levels of both BE3 and HF-BE3 and were used for expression of base editors. (b): Purification of expressed base editor proteins. Placing the His_6 tag on the C-terminus of the base editors lead to production of a truncation product for both BE3 and HF-BE3 (lanes 1 and 2). Unexpectedly, this truncation product was removed by placing the His_6 tag on the N-terminus of the protein (lanes 3-6). Inducing expression of base editors at a cell density of OD_{600} = 0.7 (lanes 4-5), later than is optimal for Cas9 expression (OD_{600} = 0.4)[1], improves yield of base editor proteins. Purification was performed using a manual HisPur resin column followed by cation exchange FPLC (Akta). **(c):** Purified BE3 and HF-BE3. Different concentrations of purified BE3 and HF-BE3 were denatured using heat and LDS and loaded onto a polyacrylamide gel. Protein samples are representative of proteins used in this study. Gels in (a), (b), and (c) are BOLT Bis-Tris Plus 4-12% polyacrylamide (Thermo Fisher). Electrophoresis and staining were performed as described in Methods.

a

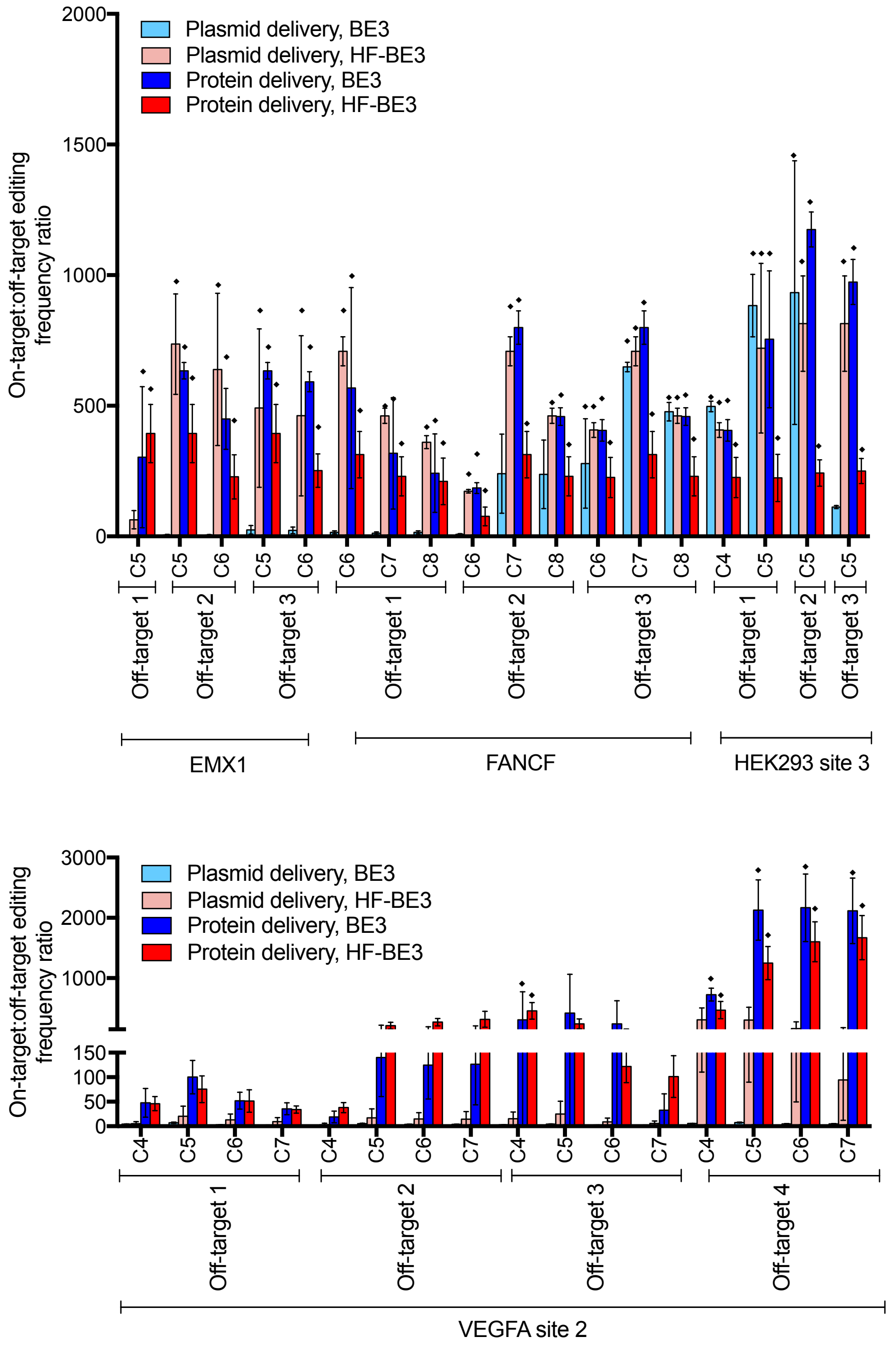

Supplementary Figure 2**: On-target:off-target base editing frequency ratios for plasmid and protein delivery of BE3 and HF-BE3.** Base editing on-target:off-target specificity ratios were calculated by dividing the on-target editing percentage at a particular cytosine in the activity window by the off-target editing percentage at the corresponding cytosine for the indicated off-target locus (see Methods). When off-target editing was below the threshold of detection (0.025% of sequencing reads), we set the off-target editing to the limit of detection (0.025%) and divided the on-target editing percentage by this upper limit. In these cases, denoted by ♦, the specificity ratios shown represent lower limits. Specificity ratios are shown for non-repetitive sgRNAs FANCF, HEK 293 site 3, and FANCF (a) and for the highly repetitive sgRNA VEGFA site 2 (b). Values and error bars reflect mean ± S.D. of three independent biological replicates performed on different days.

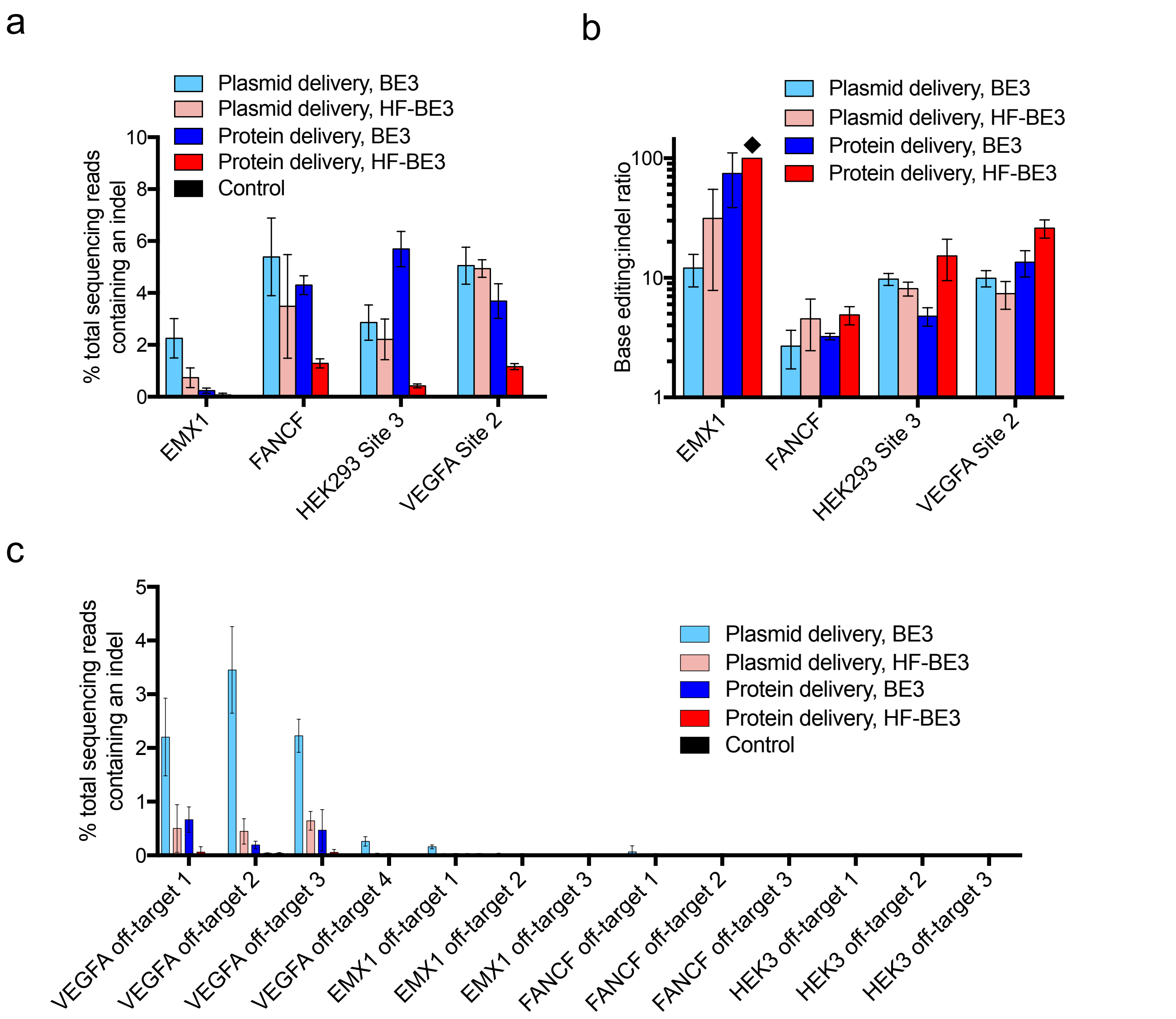

Supplementary Figure 3: **Indel formation associated with base editing at genomic loci.** (a): Indel frequency at on-target loci for VEGFA site 2, EMX1, FANCF, and HEK293 site 3 sgRNAs. (b): Ratio of base editing:indel formation. The diamond (♦) indicates no indels were detected (no significant difference in indel frequency in the treated sample and in the untreated control). (c): Indels observed at the off-target loci associated with the on-target sites interrogated in (a). Values and error bars reflect mean ± S.D. of three independent biological replicates performed on different days.

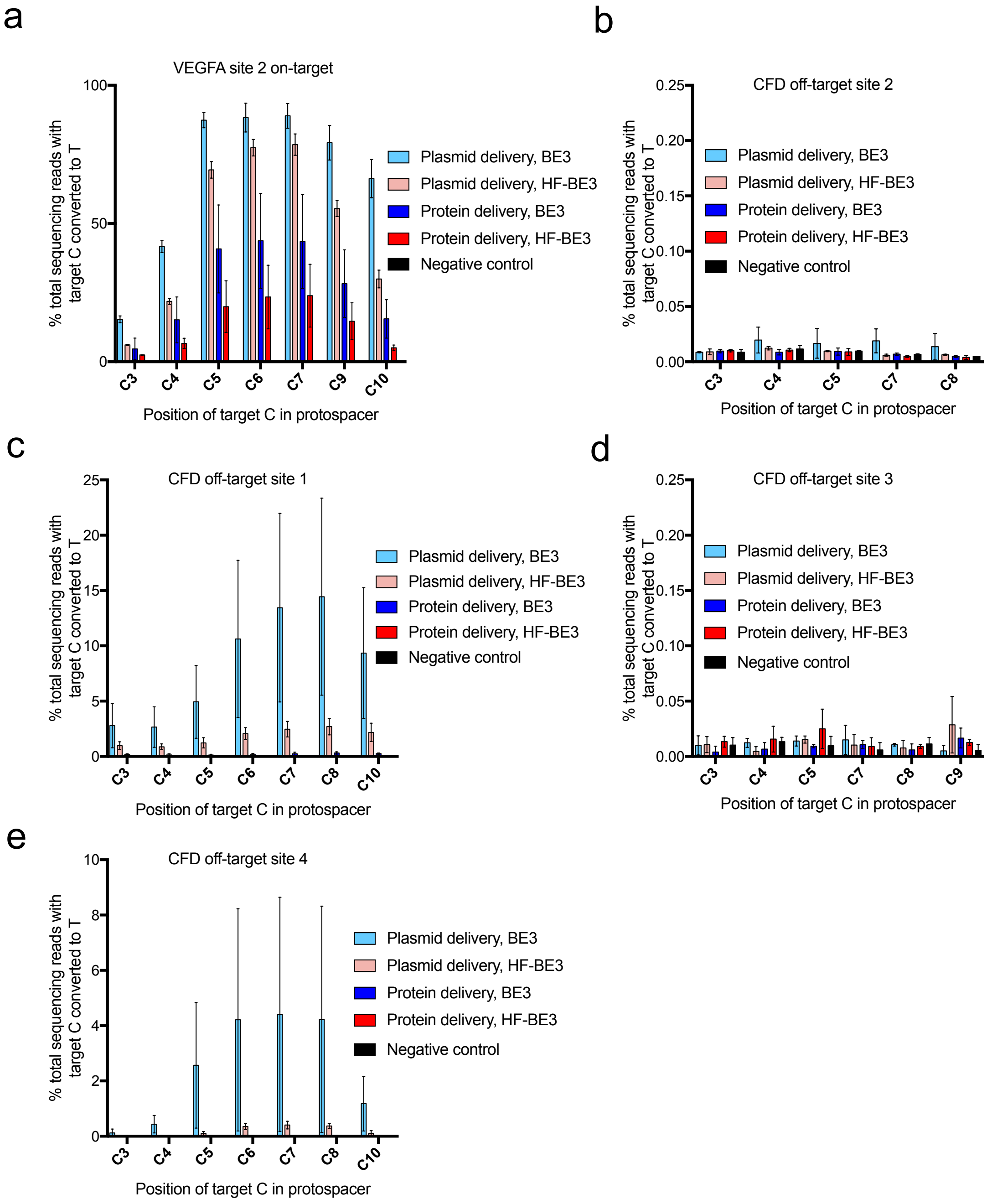

Supplementary Figure 4: **On- and off-target base editing in murine NIH/3T3 cells.** (a): On-target base editing associated with the ‘VEGFA site 2’ sgRNA (See Figure 5E for sequences). The negative control corresponds to cells treated with plasmid encoding BE3 but no sgRNA. Values and error bars reflect mean ± S.D. of three independent biological replicates performed on different days. (B-E): Off-target editing associated with this site was measured using high-throughput DNA sequencing at the top four predicted off-target loci for this sgRNA (sequences shown in Figure 5E). (b): off-target 2, (c): off-target 1, (d): off-target 3, (e): off-target 4. Values and error bars reflect mean ± S.D. of three independent biological replicates performed on different days.

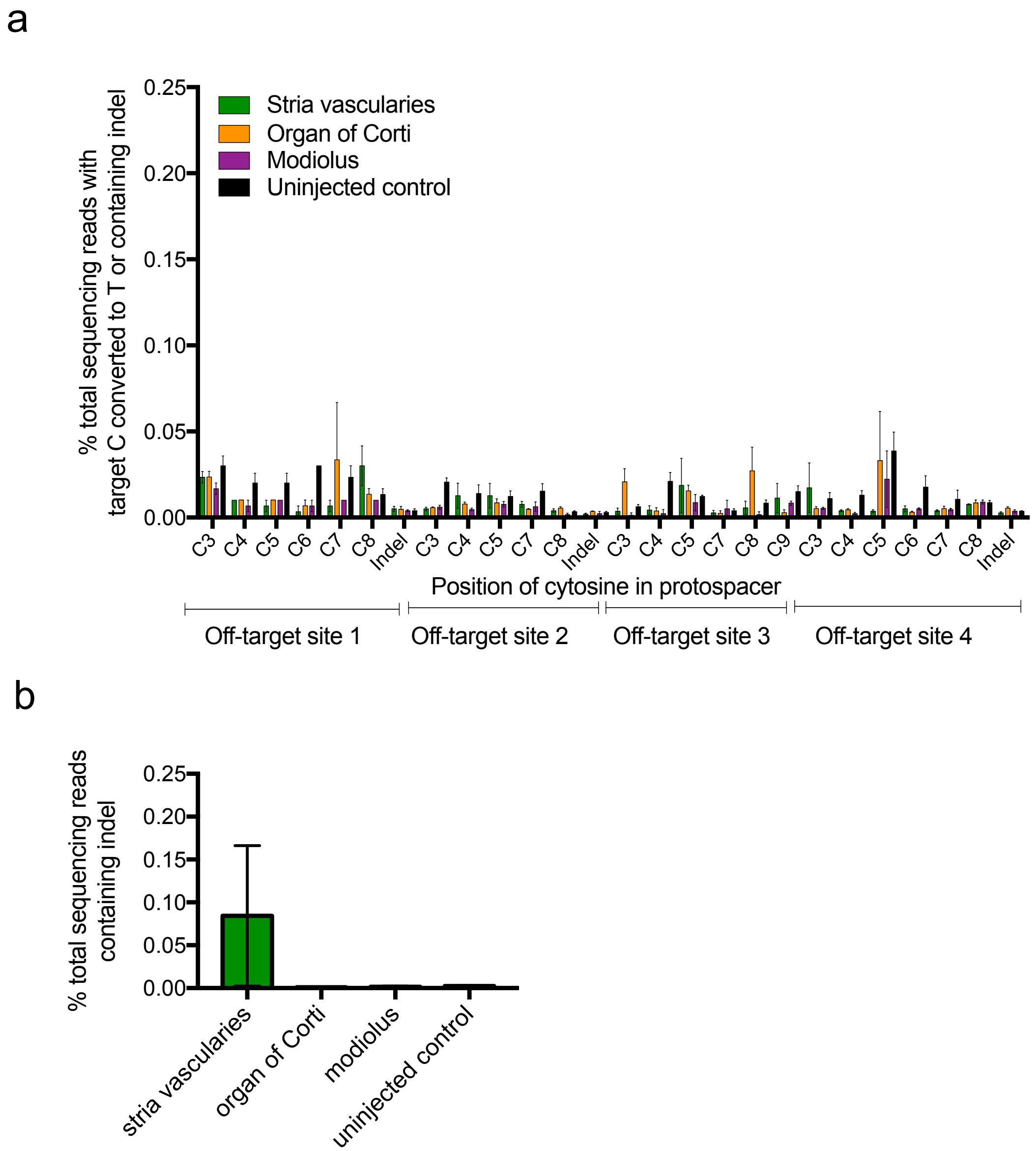

Supplementary Figure 5: **Off-target base editing and on-target indel analysis from *in vivo*-edited murine tissue.** (a): Editing is plotted for each cytosine in the base editing window of off-target loci associated with VEGFA site 2. (b): Indel rates at the on-target base editor locus. Values and error bars reflect mean ± S.E.M of three injected and three control mice.

Supplementary Note 1: **Python script used to analyze quality-filtered *in vitro*-edited DNA.**

```python
from __future__ import print_function
from __future__ import division

import Bio #This will import the BioPython suite
from Bio import SeqIO #Necessary to read/write sequence handles
from Bio.Seq import Seq
import os
import collections
import csv

inputfile = "please_specify_your_input_file_here_containing_filtered_reads" #specify the file names that contain sequences
filenames = []

for file in os.listdir(inputfile):
    if file.endswith(".fastqsanger"):
        filenames.append(file)

spacer = []
list_of_filenames = []

for file in filenames:
    site = {}
    output = open(file + ".txt", "w")
    list_of_filenames.append(file + ".txt") #allows calling of the txt files that come from fastq files later
    for rec in SeqIO.parse(file, "fastq"):
        split1=rec.seq.tostring().split("GTTCGCGGCGATCG") #14-base pair constant_region_before_protospacer
        if len(split1)>=2:
            split2=split1[1].split("TGGATCGCCTGGCA") #14-base pairc constant_region_after_protospacer
            site=split2[0]
            if len(site)==20:
                output.write(site + "\n")

BASES = 'ATGCN'
UNRECOGNIZED = 'X'
BASE_SEPERATOR = dict(zip(BASES, ',,,,\n'))
a_index = 0
t_index = 1
g_index = 2
c_index = 3
n_index = 4

def get_counts_by_column(base, count, library):
    current_count = library[count]
    if base == 'A':
        current_count[a_index] += 1
    elif base == 'T':
        current_count[t_index] += 1
    elif base == 'G':
        current_count[g_index] += 1
    elif base == 'C':
        current_count[c_index] += 1
    elif base == 'N':
        current_count[n_index] += 1

def dna_counts(list_of_sequences, sample):
    first_oligo = list_of_sequences[0]
    for i in range (len(first_oligo)):
```

```
        sample.append([0,0,0,0,0])
    for j in range(len(first_oligo)):
        for i in range(len(list_of_sequences)):
            get_counts_by_column(list_of_sequences[i][j], j, libname)


for file in list_of_filenames:
    spacer_list = open(file).read().splitlines()
    output2=[]
    dna_counts(spacer_list, output2)
    with open(file  + ".csv", "wb") as f:
        writer = csv.writer(f)
        writer.writerows(output2)
```

Supplementary Note 2**: Sequences of proteins used in this study**

Protein sequence of expressed BE3

MGSSHHHHHHSSETGPVAVDPTLRRRIEPHEFEVFFDPRELRKETCLLYEINWGGRHSIWRHTSQNTNKHVEV
NFIEKFTTERYFCPNTRCSITWFLSWSPCGECSRAITEFLSRYPHVTLFIYIARLYHHADPRNRQGLRDLISSGVTI
QIMTEQESGYCWRNFVNYSPSNEAHWPRYPHLWVRLYVLELYCIILGLPPCLNILRRKQPQLTFFTIALQSCHYQ
RLPPHILWATGLKSGSETPGTSESATPESDKKYSIGLAIGTNSVGWAVITDEYKVPSKKFKVLGNTDRHSIKKNLI
GALLFDSGETAEATRLKRTARRRYTRRKNRICYLQEIFSNEMAKVDDSFFHRLEESFLVEEDKKHERHPIFGNIV
DEVAYHEKYPTIYHLRKKLVDSTDKADLRLIYLALAHMIKFRGHFLIEGDLNPDNSDVDKLFIQLVQTYNQLFEENP
INASGVDAKAILSARLSKSRRLENLIAQLPGEKKNGLFGNLIALSLGLTPNFKSNFDLAEDAKLQLSKDTYDDDLD
NLLAQIGDQYADLFLAAKNLSDAILLSDILRVNTEITKAPLSASMIKRYDEHHQDLTLLKALVRQQLPEKYKEIFFDQ
SKNGYAGYIDGGASQEEFYKFIKPILEKMDGTEELLVKLNREDLLRKQRTFDNGSIPHQIHLGELHAILRRQEDFY
PFLKDNREKIEKILTFRIPYYVGPLARGNSRFAWMTRKSEETITPWNFEEVVDKGASAQSFIERMTNFDKNLPNE
KVLPKHSLLYEYFTVYNELTKVKYVTEGMRKPAFLSGEQKKAIVDLLFKTNRKVTVKQLKEDYFKKIECFDSVEIS
GVEDRFNASLGTYHDLLKIIKDKDFLDNEENEDILEDIVLTLTLFEDREMIEERLKTYAHLFDDKVMKQLKRRRYTG
WGRLSRKLINGIRDKQSGKTILDFLKSDGFANRNFMQLIHDDSLTFKEDIQKAQVSGQGDSLHEHIANLAGSPAIK
KGILQTVKVVDELVKVMGRHKPENIVIEMARENQTTQKGQKNSRERMKRIEEGIKELGSQILKEHPVENTQLQNE
KLYLYYLQNGRDMYVDQELDINRLSDYDVDHIVPQSFLKDDSIDNKVLTRSDKNRGKSDNVPSEEVVKKMKNYW
RQLLNAKLITQRKFDNLTKAERGGLSELDKAGFIKRQLVETRQITKHVAQILDSRMNTKYDENDKLIREVKVITLKS
KLVSDFRKDFQFYKVREINNYHHAHDAYLNAVVGTALIKKYPKLESEFVYGDYKVYDVRKMIAKSEQEIGKATAK
YFFYSNIMNFFKTEITLANGEIRKRPLIETNGETGEIVWDKGRDFATVRKVLSMPQVNIVKKTEVQTGGFSKESILP
KRNSDKLIARKKDWDPKKYGGFDSPTVAYSVLVVAKVEKGKSKKLKSVKELLGITIMERSSFEKNPIDFLEAKGY
KEVKKDLIIKLPKYSLFELENGRKRMLASAGELQKGNELALPSKYVNFLYLASHYEKLKGSPEDNEQKQLFVEQH
KHYLDEIIEQISEFSKRVILADANLDKVLSAYNKHRDKPIREQAENIIHLFTLTNLGAPAAFKYFDTTIDRKRYTSTKE
VLDATLIHQSITGLYETRIDLSQLGGDSGGSTNLSDIIEKETGKQLVIQESILMLPEEVEEVIGNKPESDILVHTAYD
ESTDENVMLLTSDAPEYKPWALVIQDSNGENKIKMLSGGSPKKKRKV

Protein sequence of expressed HF-BE3

MGSSHHHHHHSSETGPVAVDPTLRRRIEPHEFEVFFDPRELRKETCLLYEINWGGRHSIWRHTSQNTNKHVEV
NFIEKFTTERYFCPNTRCSITWFLSWSPCGECSRAITEFLSRYPHVTLFIYIARLYHHADPRNRQGLRDLISSGVTI
QIMTEQESGYCWRNFVNYSPSNEAHWPRYPHLWVRLYVLELYCIILGLPPCLNILRRKQPQLTFFTIALQSCHYQ
RLPPHILWATGLKSGSETPGTSESATPESDKKYSIGLAIGTNSVGWAVITDEYKVPSKKFKVLGNTDRHSIKKNLI
GALLFDSGETAEATRLKRTARRRYTRRKNRICYLQEIFSNEMAKVDDSFFHRLEESFLVEEDKKHERHPIFGNIV
DEVAYHEKYPTIYHLRKKLVDSTDKADLRLIYLALAHMIKFRGHFLIEGDLNPDNSDVDKLFIQLVQTYNQLFEENP
INASGVDAKAILSARLSKSRRLENLIAQLPGEKKNGLFGNLIALSLGLTPNFKSNFDLAEDAKLQLSKDTYDDDLD
NLLAQIGDQYADLFLAAKNLSDAILLSDILRVNTEITKAPLSASMIKRYDEHHQDLTLLKALVRQQLPEKYKEIFFDQ
SKNGYAGYIDGGASQEEFYKFIKPILEKMDGTEELLVKLNREDLLRKQRTFDNGSIPHQIHLGELHAILRRQEDFY
PFLKDNREKIEKILTFRIPYYVGPLARGNSRFAWMTRKSEETITPWNFEEVVDKGASAQSFIERMTAFDKNLPNE
KVLPKHSLLYEYFTVYNELTKVKYVTEGMRKPAFLSGEQKKAIVDLLFKTNRKVTVKQLKEDYFKKIECFDSVEIS
GVEDRFNASLGTYHDLLKIIKDKDFLDNEENEDILEDIVLTLTLFEDREMIEERLKTYAHLFDDKVMKQLKRRRYTG
WGALSRKLINGIRDKQSGKTILDFLKSDGFANRNFMALIHDDSLTFKEDIQKAQVSGQGDSLHEHIANLAGSPAIK
KGILQTVKVVDELVKVMGRHKPENIVIEMARENQTTQKGQKNSRERMKRIEEGIKELGSQILKEHPVENTQLQNE
KLYLYYLQNGRDMYVDQELDINRLSDYDVDHIVPQSFLKDDSIDNKVLTRSDKNRGKSDNVPSEEVVKKMKNYW
RQLLNAKLITQRKFDNLTKAERGGLSELDKAGFIKRQLVETRAITKHVAQILDSRMNTKYDENDKLIREVKVITLKS
KLVSDFRKDFQFYKVREINNYHHAHDAYLNAVVGTALIKKYPKLESEFVYGDYKVYDVRKMIAKSEQEIGKATAK
YFFYSNIMNFFKTEITLANGEIRKRPLIETNGETGEIVWDKGRDFATVRKVLSMPQVNIVKKTEVQTGGFSKESILP
KRNSDKLIARKKDWDPKKYGGFDSPTVAYSVLVVAKVEKGKSKKLKSVKELLGITIMERSSFEKNPIDFLEAKGY
KEVKKDLIIKLPKYSLFELENGRKRMLASAGELQKGNELALPSKYVNFLYLASHYEKLKGSPEDNEQKQLFVEQH
KHYLDEIIEQISEFSKRVILADANLDKVLSAYNKHRDKPIREQAENIIHLFTLTNLGAPAAFKYFDTTIDRKRYTSTKE
VLDATLIHQSITGLYETRIDLSQLGGDSGGSTNLSDIIEKETGKQLVIQESILMLPEEVEEVIGNKPESDILVHTAYD
ESTDENVMLLTSDAPEYKPWALVIQDSNGENKIKMLSGGSPKKKRKV

Supplementary Note 3: **Sequences of oligonucleotides used in this study**

Unpublished Primers used to amplify off target genomic DNA for HTS in human cells

fwd_VEGFA_site2_off_target_1_human	ACACTCTTTCCCTACACGACGCTCTTCCGATCTNNNNTCCTACAAGTAACAGTCCAAGAA
rev_VEGFA_site2_off_target_1_human	TGGAGTTCAGACGTGTGCTCTTCCGATCTTTCTGCAACTTAACTTACGTGAAA
fwd_VEGFA_site2_off_target_2_human	ACACTCTTTCCCTACACGACGCTCTTCCGATCTNNNNACCAAGCCCATTTGTCCAGG
rev_VEGFA_site2_off_target_2_human	TGGAGTTCAGACGTGTGCTCTTCCGATCTTCCTTCTTTTTGAGCTTTGGGC
fwd_VEGFA_site2_off_target_3_human	ACACTCTTTCCCTACACGACGCTCTTCCGATCTNNNNTCCATACCAGCAGCAGTTCC
rev_VEGFA_site2_off_target_3_human	TGGAGTTCAGACGTGTGCTCTTCCGATCTCTCACCTCAGCTCCTGCAC
fwd_VEGFA_site2_off_target_4_human	ACACTCTTTCCCTACACGACGCTCTTCCGATCTNNNNCCCACTGATTCTACACCATGGT
rev_VEGFA_site2_off_target_4_human	TGGAGTTCAGACGTGTGCTCTTCCGATCTGGAGTTCCCAACCTTTTTGACA

Other primers (for off target sites associated with HEK_3, EMX1, FANCF) were previously published

Primers used to amplify off target genomic DNA for HTS in murine cells

fwd_VEGFA_site2_off_target_1_murine	ACACTCTTTCCCTACACGACGCTCTTCCGATCTNNNNCTGGCTGGAGATTCAGAGACAC
rev_VEGFA_site2_off_target_1_murine	TGGAGTTCAGACGTGTGCTCTTCCGATCTCTGCCCCTTCTGACACACATAC
fwd_VEGFA_site2_off_target_2_murine	ACACTCTTTCCCTACACGACGCTCTTCCGATCTNNNNACCCCTCAAGGCTTGACATTTC
rev_VEGFA_site2_off_target_2_murine	TGGAGTTCAGACGTGTGCTCTTCCGATCTTGAAAAGTTGGGAGAGGGGATG
fwd_VEGFA_site2_off_target_3_murine	ACACTCTTTCCCTACACGACGCTCTTCCGATCTNNNNTTTGTACCCCAGTCCCCTCATC
rev_VEGFA_site2_off_target_3_murine	TGGAGTTCAGACGTGTGCTCTTCCGATCTTGAAGTTACGGGGATGTCACTTG
fwd_VEGFA_site2_off_target_4_murine	ACACTCTTTCCCTACACGACGCTCTTCCGATCTNNNNTTAACATCCAGTCTCCCAAACACA
rev_VEGFA_site2_off_target_4_murine	TGGAGTTCAGACGTGTGCTCTTCCGATCTACACACACACACTACTAGGACA

Primers used to amplify on target genomic DNA for HTS in murine cells

fwd_VEGFA_site2_on_target_murine	ACACTCTTTCCCTACACGACGCTCTTCCGATCTNNNNCGCTACTACGGAGCGAGAAG
rev_VEGFA_site2_on_target_murine	TGGAGTTCAGACGTGTGCTCTTCCGATCTACAGGGGCAAAGTGAGTGAC

Primers used for generating PCR products to serve as substrates for T7 transcription of sgRNAs

rev_sgRNA_T7: used in all cases	AAAAAAAGCACCGACTCGGTGCCAC
fwd_sgRNA_T7_EMX1	TAATACGACTCACTATAGGGAGTCCGAGCAGAAGAAGAAGTTTTAGAGCTAGAAATAGCA
fwd_sgRNA_T7_FANCF	TAATACGACTCACTATAGGGGAATCCCTTCTGCAGCACCGTTTTAGAGCTAGAAATAGCA
fwd_sgRNA_T7_HEK_site_3	TAATACGACTCACTATAGGGGCCCAGACTGAGCACGTGAGTTTTAGAGCTAGAAATAGCA
fwd_sgRNA_T7_VEGFA_site_2	TAATACGACTCACTATAG GACCCCCTCCACCCCGCCTCGTTTTAGAGCTAGAAATAGCA

fwd_sgRNA_T7_TC_repeat_in_vitro	TAATACGACTCACTATAGGTCTCTCTCTCTCTCTCTCTCGTTTTAGAGCTAGAAATAGCA

Primers used for generating sgRNA transfection plasmids

The pFYF1320 plasmid was used as template as previously described (Komor et al). The sequence of other sgRNA plasmids was previously reported

rev_sgRNA_plasmid	GGTGTTTCGTCCTTTCCACAAG
fwd_VEGFA_site_2	GACCCCCTCCACCCCGCCTCGTTTTAGAGCTAGAAATAGCAAGTTAAAATAAGGC

Sequences of ssDNA substrates used in in vitro deaminase assays

fwd_TC_repeat_substrate	ACGTAAACGGCCACAAGTTCGCGGCGATCGTCTCTCTCTCTCTCTCTCTCTGGATCGCCTGGCATCTTCTTCAAGGACG
rev_TC_repeat_substrate	CGTCCTTGAAGAAGATGCCAGGCGATCCAGAGAGAGAGAGAGAGAGAGAGACGATCGCCGCGAACTTGTGGCCGTTTACGT

Previously published primers used to amplify off target genomic DNA for HTS in human cells

fwd_EMX1_HTS	ACACTCTTTCCCTACACGACGCTCTTCCGATCTNNNNCAGCTCAGCCTGAGTGTTGA
rev_EMX1_HTS	TGGAGTTCAGACGTGTGCTCTTCCGATCTCTCGTGGGTTTGTGGTTGC
fwd_FANCF_HTS	ACACTCTTTCCCTACACGACGCTCTTCCGATCTNNNNCATTGCAGAGAGGCGTATCA
rev_FANCF_HTS	TGGAGTTCAGACGTGTGCTCTTCCGATCTGGGGTCCCAGGTGCTGAC
fwd_HEK293_site3_HTS	ACACTCTTTCCCTACACGACGCTCTTCCGATCTNNNNATGTGGGCTGCCTAGAAAGG
rev_HEK293_site3_HTS	TGGAGTTCAGACGTGTGCTCTTCCGATCTCCCAGCCAAACTTGTCAACC
fwd_EMX1_off1_HTS	ACACTCTTTCCCTACACGACGCTCTTCCGATCTNNNNAGTAGCCTCTTTCTCAATGTGC
rev_EMX1_off1_HTS	TGGAGTTCAGACGTGTGCTCTTCCGATCTGCTTTCACAAGGATGCAGTCT
fwd_EMX1_off2_HTS	ACACTCTTTCCCTACACGACGCTCTTCCGATCTNNNNGAGCTAGACTCCGAGGGGA
rev_EMX1_off2_HTS	TGGAGTTCAGACGTGTGCTCTTCCGATCTTCCTCGTCCTGCTCTCACTT
fwd_EMX1_off3_HTS	ACACTCTTTCCCTACACGACGCTCTTCCGATCTNNNNAGAGGCTGAAGAGGAAGACCA
rev_EMX1_off3_HTS	TGGAGTTCAGACGTGTGCTCTTCCGATCTGGCCCAGCTGTGCATTCTAT
fwd_FANCF_off1_HTS	ACACTCTTTCCCTACACGACGCTCTTCCGATCTNNNNAACCCACTGAAGAAGCAGGG
rev_FANCF_off1_HTS	TGGAGTTCAGACGTGTGCTCTTCCGATCTGGTGCTTAATCCGGCTCCAT
fwd_FANCF_off2_HTS	ACACTCTTTCCCTACACGACGCTCTTCCGATCTNNNNTCCAGTGTTTCCATCCCGAA
rev_FANCF_off2_HTS	TGGAGTTCAGACGTGTGCTCTTCCGATCTCCTCTGACCTCCACAACTCT
fwd_FANCF_off3_HTS	ACACTCTTTCCCTACACGACGCTCTTCCGATCTNNNNCTGGGTACAGTTCTGCGTGT
rev_FANCF_off3_HTS	TGGAGTTCAGACGTGTGCTCTTCCGATCTTCACTCTGAGCATCGCCAAG
fwd_HEK293_site3_off1_HTS	ACACTCTTTCCCTACACGACGCTCTTCCGATCTNNNNTCCCCTGTTGACCTGGAGAA

rev_HEK293_site3_off1_HTS	TGGAGTTCAGACGTGTGCTCTTCCGATCTCACTGTACTTGCCCTGACCA
fwd_HEK293_site3_off2_HTS	ACACTCTTTCCCTACACGACGCTCTTCCGATCTNNNNTTGGTGTTGACAGGGAGCAA
rev_HEK293_site3_off2_HTS	TGGAGTTCAGACGTGTGCTCTTCCGATCTCTGAGATGTGGGCAGAAGGG
fwd_HEK293_site3_off3_HTS	ACACTCTTTCCCTACACGACGCTCTTCCGATCTNNNNTGAGAGGGAACAGAAGGGCT
rev_HEK293_site3_off3_HTS	TGGAGTTCAGACGTGTGCTCTTCCGATCTGTCCAAAGGCCCAAGAACCT

Primers used to amplify on target genomic DNA for HTS in zebrafish

fwd_TYR1_zebrafish	ACACTCTTTCCCTACACGACGCTCTTCCGATCTNNNNGTTCCCCCGAGTCTGCACCT
rev_TYR1_zebrafish	TGGAGTTCAGACGTGTGCTCTTCCGATCTCGAACTTGCATTCGCCGCAA
fwd_TYR2_zebrafish	ACACTCTTTCCCTACACGACGCTCTTCCGATCTNNNNTTCTGCCTTGGCATCGGGTG
rev_TYR2_zebrafish	TGGAGTTCAGACGTGTGCTCTTCCGATCTCACCATACCGCCCCTAGAACTAACATTC
fwd_TYR3_zebrafish	ACACTCTTTCCCTACACGACGCTCTTCCGATCTNNNNACAACTGCTTTCCATGGTGTGT
rev_TYR3_zebrafish	TGGAGTTCAGACGTGTGCTCTTCCGATCTTCCCAGGGCTTTCGTGGAGA

Locus and cytosine position	*P* values (Student's two-tailed t-test) for comparisons between listed treatments									
	plasmid BE3 vs plasmid HF-BE3	plasmid BE3 vs protein BE3	plasmid BE3 vs protein HF-BE3	plasmid BE3 vs control	plasmid HF-BE3 vs protein BE3	plasmid HF-BE3 vs protein HF-BE3	plasmid HF-BE3 vs control	protein BE3 vs protein HF-BE3	protein BE3 vs control	protein HF-BE3 vs control
EMX1, C5	0.053	0.000	0.001	0.000	0.416	0.056	0.003	0.023	0.000	0.004
EMX1, C6	0.065	0.000	0.000	0.000	0.445	0.023	0.004	0.001	0.000	0.003
FANCF, C6	0.152	0.017	0.003	0.000	0.137	0.003	0.000	0.002	0.000	0.004
FANCF, C7	0.591	0.554	0.007	0.000	0.914	0.007	0.000	0.008	0.000	0.006
FANCF, C8	0.011	0.026	0.004	0.000	0.958	0.018	0.000	0.023	0.000	0.007
FANCF, C11	0.524	0.948	0.019	0.001	0.363	0.010	0.000	0.010	0.000	0.021
HEK site 3, C3	0.061	0.001	0.071	0.000	0.002	0.002	0.005	0.00199	0.001	0.003
HEK site 3, C4	0.048	0.924	0.010	0.004	0.001	0.001	0.001	0.00004	0.000	0.001
HEK site 3, C5	0.291	0.592	0.016	0.006	0.243	0.243	0.002	0.00022	0.000	0.001
VEGFA site 2, C3	0.060	0.416	0.239	0.010	0.042	0.280	0.002	0.475	0.002	0.018
VEGFA site 2, C4	0.036	0.191	0.047	0.004	0.032	0.803	0.002	0.066	0.000	0.005
VEGFA site 2, C5	0.098	0.650	0.028	0.003	0.044	0.169	0.002	0.004	0.000	0.001
VEGFA site 2, C6	0.452	0.781	0.118	0.004	0.165	0.239	0.002	0.013	0.000	0.001
VEGFA site 2, C7	0.401	0.683	0.172	0.003	0.120	0.454	0.002	0.026	0.000	0.001
VEGFA site 2, C9	0.225	0.308	0.254	0.004	0.504	0.828	0.004	0.624	0.000	0.002
VEGFA site 2, C10	0.064	0.061	0.023	0.005	0.732	0.257	0.009	0.057	0.000	0.003
EMX1, C5 off-target 1	0.003	0.003	0.002	0.002	0.119	0.036	0.035	0.269	0.255	0.643
EMX1, C5 off-target 2	0.013	0.013	0.013	0.013	0.158	0.294	0.521	0.390	0.058	0.054
EMX1, C6 off-target 2	0.024	0.024	0.024	0.024	0.285	0.560	0.954	0.420	0.103	0.306
EMX1, C5 off-target 3	0.022	0.019	0.019	0.019	0.297	0.297	0.300	>0.99999	0.882	0.815
EMX1, C6 off-target 3	0.017	0.015	0.015	0.015	0.296	0.296	0.328	>0.99999	0.051	0.025
FANCF, C5 off-target 1	0.031	0.031	0.031	0.031	0.314	0.530	0.333	0.337	0.349	0.618
FANCF, C6 off-target 1	0.016	0.016	0.016	0.016	0.347	0.786	0.930	0.338	0.344	0.678
FANCF, C7 off-target 1	0.028	0.028	0.028	0.027	0.374	0.039	0.106	0.353	0.346	0.639
FANCF, C8 off-target 1	0.014	0.014	0.014	0.014	0.341	0.932	0.685	0.343	0.318	0.605
FANCF, C11 off-target 1	0.007	0.007	0.007	0.007	0.374	0.001	0.000	>0.99999	0.475	0.016
FANCF, C6 off-target 2	0.099	0.099	0.032	0.036	0.599	0.475	0.912	0.914	0.393	0.060
FANCF, C7 off-target 2	0.080	0.080	0.027	0.030	0.898	0.638	0.819	0.539	0.859	0.530
FANCF, C8 off-target 2	0.123	0.123	0.045	0.050	0.789	0.538	0.960	0.047	0.539	0.252
FANCF, C10 off-target 2	0.093	0.093	0.029	0.033	0.630	0.509	0.847	0.768	0.670	0.482
FANCF, C11 off-target 2	0.264	0.264	0.127	0.107	0.599	0.658	0.326	>0.99999	0.047	0.345
FANCF, C6 off-target 3	0.872	0.872	0.492	0.108	0.239	0.493	0.129	0.469	0.584	0.252
FANCF, C7 off-target 3	>0.99999	>0.99999	0.859	0.016	0.537	0.866	0.116	0.572	0.272	0.595
FANCF, C8 off-target 3	0.886	0.886	0.246	0.757	>0.99999	0.001	0.648	0.495	0.780	0.650
FANCF, C10 off-target 3	0.566	0.566	0.284	0.202	0.053	0.387	0.260	0.453	0.913	0.541
FANCF, C11 off-target 3	0.422	0.422	0.145	0.145	0.495	0.230	0.230	0.658	0.658	>0.99999
HEK293 site 3, C3 off-target 1	>0.99999	0.910	0.412	0.326	0.910	0.412	0.326	0.293	0.223	0.480
HEK293 site 3, C4 off-target 1	>0.99999	0.994	0.437	0.391	0.994	0.437	0.391	0.495	0.451	0.614
HEK293 site 3, C5 off-target 1	>0.99999	0.616	0.814	0.337	0.616	0.814	0.337	0.459	0.116	0.481
HEK293 site 3, C3 off-target 2	0.285	0.473	0.100	0.473	0.141	0.735	0.141	0.038	>0.99999	0.038
HEK293 site 3, C5 off-target 2	0.375	0.294	0.177	0.294	0.687	0.428	0.687	0.064	>0.99999	0.064
HEK293 site 3, C9 off-target 2	0.053	0.624	0.374	0.624	0.554	0.154	0.554	0.872	>0.99999	0.872
HEK293 site 3, C3 off-target 3	0.067	0.116	0.768	0.435	0.519	0.230	0.561	0.349	0.768	0.643
HEK293 site 3, C5 off-target 3	0.011	0.011	0.011	0.011	0.016	0.643	0.435	0.184	0.025	0.346
HEK293 site 3, C9 off-target 3	>0.99999	0.374	0.652	0.811	0.132	0.539	0.776	0.609	0.643	0.893
VEGFA site 2, C4 off-target 1	0.101	0.015	0.014	0.012	0.041	0.032	0.025	0.117	0.012	0.001
VEGFA site 2, C5 off-target 1	0.060	0.013	0.012	0.010	0.078	0.062	0.044	0.201	0.009	0.012
VEGFA site 2, C6 off-target 1	0.019	0.005	0.005	0.004	0.080	0.062	0.045	0.087	0.002	0.012
VEGFA site 2, C7 off-target 1	0.017	0.004	0.004	0.003	0.080	0.060	0.037	0.076	0.001	0.002
VEGFA site 2, C9 off-target 1	0.230	0.088	0.037	0.011	0.667	0.256	0.051	0.134	0.004	0.007
VEGFA site 2, C10 off-target 1	0.535	0.136	0.106	0.035	0.283	0.211	0.050	0.717	0.028	0.010
VEGFA site 2, C4 off-target 2	0.038	0.004	0.003	0.003	0.087	0.051	0.048	0.063	0.048	0.134
VEGFA site 2, C5 off-target 2	0.033	0.004	0.004	0.004	0.078	0.061	0.059	0.028	0.020	0.248
VEGFA site 2, C6 off-target 2	0.026	0.005	0.005	0.004	0.051	0.038	0.038	0.043	0.038	0.783
VEGFA site 2, C7 off-target 2	0.053	0.006	0.005	0.005	0.072	0.056	0.055	0.078	0.064	0.704
VEGFA site 2, C8 off-target 2	0.071	0.006	0.006	0.006	0.079	0.065	0.065	0.118	0.107	0.703
VEGFA site 2, C9 off-target 2	0.193	0.008	0.007	0.006	0.103	0.090	0.084	0.068	0.007	0.217
VEGFA site 2, C10 off-target 2	0.063	0.003	0.003	0.002	0.116	0.107	0.090	0.545	0.016	0.346
VEGFA site 2, C4 off-target 3	0.005	0.003	0.003	0.003	0.091	0.031	0.030	0.116	0.107	0.158
VEGFA site 2, C5 off-target 3	0.011	0.007	0.005	0.005	0.211	0.048	0.042	0.220	0.177	0.001
VEGFA site 2, C6 off-target 3	0.020	0.005	0.003	0.003	0.142	0.038	0.033	0.193	0.149	0.015
VEGFA site 2, C7 off-target 3	0.045	0.006	0.003	0.003	0.101	0.035	0.030	0.093	0.060	0.083
VEGFA site 2, C8 off-target 3	0.069	0.007	0.005	0.005	0.087	0.045	0.039	0.120	0.067	0.041
VEGFA site 2, C9 off-target 3	0.093	0.006	0.005	0.005	0.041	0.032	0.028	0.396	0.195	0.005
VEGFA site 2, C10 off-target 3	0.342	0.011	0.008	0.007	0.109	0.081	0.069	0.273	0.098	0.036
VEGFA site 2, C3 off-target 4	0.001	0.001	0.001	0.001	0.374	0.374	0.230	0.271	0.358	0.633
VEGFA site 2, C4 off-target 4	0.007	0.006	0.006	0.006	0.137	0.137	0.137	0.592	0.862	0.690
VEGFA site 2, C5 off-target 4	0.007	0.007	0.007	0.007	0.026	0.017	0.018	0.461	0.655	0.279
VEGFA site 2, C6 off-target 4	0.005	0.004	0.004	0.004	0.021	0.018	0.018	0.398	0.546	0.149
VEGFA site 2, C7 off-target 4	0.007	0.006	0.006	0.006	0.051	0.048	0.050	0.373	0.720	0.029
VEGFA site 2, C8 off-target 4	0.007	0.006	0.006	0.006	0.092	0.092	0.092	0.325	0.014	0.275
VEGFA site 2, C9 off-target 4	0.016	0.007	0.007	0.007	0.150	0.150	0.150	0.502	1.000	0.615
VEGFA site 2, C10 off-target 4	0.213	0.009	0.009	0.009	0.261	0.261	0.261	0.653	0.575	0.660

Supplementary Table 1: ***P*-values for differences in base editing under different treatment conditions at all loci evaluated in this study.** *p*-values were calculated using the Student's two tailed t-test as described in the Materials and Methods. When the p-value indicated a significant difference ($p < 0.05$), the corresponding entry has been highlighted in red.

Site	Sequence	GUIDE-Seq count
EMX1 on-target	GAGTC_5C_6GAGCAGAAGAAGAAGGG	4,521
EMX1 off-target 1	GAGTC_5TAAGCAGAAGAAGAAGAG	1,445
EMX1 off-target 2	GAGGC_5C_6GAGCAGAAGAAAGACGG	700
EMX1 off-target 3	GAGTC_5C_6TAGCAGGAGAAGAAGAG	390
HEK293 site 3 on-target	GGCC_4C_5AGACTGAGCACGTGATGG	2,074
HEK293 site 3 off-target 1	CACC_4C_5AGACTGAGCACGTGCTGG	327
HEK293 site 3 off-target 2	GACAC_5AGACTGGGCACGTGAGGG	306
HEK293 site 3 off-target 3	AGCTC_5AGACTGAGCAAGTGAGGG	136
VEGFA site 2 on-target	GA$C_3C_4C_5C_6C_7$TC_9C_{10}ACCCCGCCTCCGG	540
VEGFA site 2 off-target 1	CTA$C_4C_5C_6C_7$TC_9C_{10}ACCCCGCCTCCGG	1,925
VEGFA site 2 off-target 2	ATT$C_4C_5C_6C_7C_8C_9C_{10}$ACCCCGCCTCAGG	1,549
VEGFA site 2 off-target 3	ACA$C_4C_5C_6C_7C_8C_9C_{10}$ACCCCGCCTCAGG	1,178
VEGFA site 2 off-target 4	TG$C_3C_4C_5C_6C_7C_8C_9C_{10}$ACCCCACCTCTGG	1,107
FANCF on-target	GGAAT$C_6C_7C_8$TTC_{11}TGCAGCACCTGG	4,816
FANCF off-target 1	GGAA$C_5C_6C_7C_8$GTC_{11}TGCAGCACCAGG	2,099
FANCF off-target 2	GGAGT$C_6C_7C_8$T$C_{10}C_{11}$TACAGCACCAGG	524
FANCF off-target 3	AGAGG$C_6C_7C_8C_9$TC_{11}TGCAGCACCAGG	150

Supplementary Table 2**: Protospacer and PAM sequences for the on- and off-target human genomic loci studied in this work.** The off-target sites were chosen based on their GUIDE-Seq read count [2]. Cytosines within the editing window for a particular sgRNA are shown in red and numbered. The PAM sequence is shown in blue. Protospacer bases in off-target loci that differ from their respective on-target loci have been underlined. For genomic sequences interrogated in murine samples, see Figure 5E.

Site	Sequence	CFD score	Description of locus
On-target	GACCCCCTCCACCCCGCCTCCGG		VEGFA site 2
Off-target 1	TCCCCCCTCCACCCCACCTCCGG	0.7857	intergenic:mmu-mir-21c-Nrp1/Mir1903
Off-target 2	TGCCCACCTCACCCCGCCTCTGG	0.65	intron:Vipr1
Off-target 3	GCCCCTCCCAACCCCACCTCTGG	0.6323	intron:Nos1ap
Off-target 4	CACCCCCCTCACCCCGCCTCAGG	0.625	intergenic:Unc5b-mmu-mir-6408

Supplementary Table 3: **Protospacer and PAM sequences for the predicted off-target loci in the mouse genome associated with the VEGFA site 2 sgRNA.** CFD scores [3] were calculated using CRISPOR [4]. Positions in the off-target protospacers that differ from the on-target sequence are underlined.

Site	Sequence
TYR1	GTC$_3$AGGTC$_8$GAGGGTTCTGTCAGG
TYR2	CTTC$_4$C$_5$AGGATGAGAACACAGAGG
TYR3	CAAC$_4$C$_5$AC$_7$TGCTCAAAGATGCTGG

Supplementary Table 4: **Protospacer and PAM sequences for the zebrafish genomic loci studied in this work.**

HEK cell samples

Sample Description	Replicate 1	Replicate 2	Replicate 3	Amplicon
Protein, BE3	237256	295609	159391	VEGFA On Target
Protein, HF-BE3	383480	389874	383467	VEGFA On Target
Plasmid, BE3	315213	280891	335668	VEGFA On Target
Plasmid, HF-BE3	196323	251965	369201	VEGFA On Target
Control	390748	395523	353614	VEGFA On Target
Protein, BE3	19280	26472	24799	FANCF Off Target Site #1
Protein, HF-BE3	36383	30007	39193	FANCF Off Target Site #1
Plasmid, BE3	35580	29557	22243	FANCF Off Target Site #1
Plasmid, HF-BE3	40371	27187	28248	FANCF Off Target Site #1
Control	35106	37274	34939	FANCF Off Target Site #1
Protein, BE3	82978	124689	83840	VEGFA Off Target Site #1
Protein, HF-BE3	142404	140482	142220	VEGFA Off Target Site #1
Plasmid, BE3	112027	117071	100894	VEGFA Off Target Site #1
Plasmid, HF-BE3	114187	98876	122553	VEGFA Off Target Site #1
Control	76854	90547	89271	VEGFA Off Target Site #1
Protein, BE3	14514	25515	19325	VEGFA Off Target Site #2
Protein, HF-BE3	24678	24363	25312	VEGFA Off Target Site #2
Plasmid, BE3	16945	19918	10225	VEGFA Off Target Site #2
Plasmid, HF-BE3	12200	14769	17797	VEGFA Off Target Site #2
Control	8739	13648	7818	VEGFA Off Target Site #2
Protein, BE3	17924	111693	173909	VEGFA Off Target Site #3
Protein, HF-BE3	243899	300503	276139	VEGFA Off Target Site #3
Plasmid, BE3	208476	291370	155430	VEGFA Off Target Site #3
Plasmid, HF-BE3	117174	154033	199152	VEGFA Off Target Site #3
Control	119263	170436	121686	VEGFA Off Target Site #3
Protein, BE3	237799	262947	185371	VEGFA Off Target Site #4
Protein, HF-BE3	313253	233699	244922	VEGFA Off Target Site #4
Plasmid, BE3	243094	230316	234421	VEGFA Off Target Site #4
Plasmid, HF-BE3	170958	160091	140693	VEGFA Off Target Site #4
Control	158691	148720	137270	VEGFA Off Target Site #4
Protein, BE3	28684	28237	43315	HEK3 On Target
Protein, HF-BE3	49300	42576	57690	HEK3 On Target
Plasmid, BE3	55008	55813	54310	HEK3 On Target
Plasmid, HF-BE3	55199	11384	7659	HEK3 On Target
Control	63741	42878	48524	HEK3 On Target
Protein, BE3	104822	181792	161090	HEK3 Off Target Site # 1
Protein, HF-BE3	204580	175561	177303	HEK3 Off Target Site # 1
Plasmid, BE3	178584	152264	206863	HEK3 Off Target Site # 1
Plasmid, HF-BE3	191297	138425	160789	HEK3 Off Target Site # 1
Control	190303	190061	190516	HEK3 Off Target Site # 1
Protein, BE3	146089	95113	135015	HEK3 Off Target Site # 2
Protein, HF-BE3	155947	136541	157991	HEK3 Off Target Site # 2
Plasmid, BE3	150036	128438	158905	HEK3 Off Target Site # 2
Plasmid, HF-BE3	371077	123642	142562	HEK3 Off Target Site # 2
Control	130322	134545	141833	HEK3 Off Target Site # 2
Protein, BE3	145058	175338	161837	HEK3 Off Target Site # 3
Protein, HF-BE3	212337	178993	179887	HEK3 Off Target Site # 3
Plasmid, BE3	186452	166500	80441	HEK3 Off Target Site # 3
Plasmid, HF-BE3	163732	118453	134719	HEK3 Off Target Site # 3
Control	131461	134470	155608	HEK3 Off Target Site # 3
Protein, BE3	41986	61678	67890	FANCF On Target
Protein, HF-BE3	41057	55850	86411	FANCF On Target
Plasmid, BE3	39114	48575	70074	FANCF On Target
Plasmid, HF-BE3	41617	55638	75718	FANCF On Target
Control	68852	59422	81265	FANCF On Target
Protein, BE3	113462	80529	191344	FANCF Off Target Site # 1
Protein, HF-BE3	202662	233981	203024	FANCF Off Target Site # 1
Plasmid, BE3	208912	202044	107234	FANCF Off Target Site # 1
Plasmid, HF-BE3	86494	113989	86807	FANCF Off Target Site # 1
Control	92255	72386	56661	FANCF Off Target Site # 1
Protein, BE3	96271	117442	84374	FANCF Off Target Site # 2
Protein, HF-BE3	105624	102312	105343	FANCF Off Target Site # 2
Plasmid, BE3	101002	98747	70052	FANCF Off Target Site # 2
Plasmid, HF-BE3	308966	69787	83184	FANCF Off Target Site # 2
Control	99986	100344	100659	FANCF Off Target Site # 2
Protein, BE3	25524	182451	65388	FANCF Off Target Site # 3
Protein, HF-BE3	71858	75553	71785	FANCF Off Target Site # 3
Plasmid, BE3	60980	57360	78169	FANCF Off Target Site # 3
Plasmid, HF-BE3	68316	34659	85718	FANCF Off Target Site # 3
Control	49685	57388	60418	FANCF Off Target Site # 3
Protein, BE3	46981	90793	79439	EMX1 On Target
Protein, HF-BE3	58629	71186	61575	EMX1 On Target
Plasmid, BE3	70817	82736	75706	EMX1 On Target
Plasmid, HF-BE3	77038	71123	78511	EMX1 On Target
Control	62183	48574	68439	EMX1 On Target
Protein, BE3	165905	257565	142888	EMX 1 Off Target Site # 1
Protein, HF-BE3	148339	151300	130712	EMX 1 Off Target Site # 1
Plasmid, BE3	101950	103226	203004	EMX 1 Off Target Site # 1
Plasmid, HF-BE3	167969	175193	97010	EMX 1 Off Target Site # 1
Control	101476	150435	102327	EMX 1 Off Target Site # 1
Protein, BE3	136738	213438	118711	EMX 1 Off Target Site # 2
Protein, HF-BE3	123413	126114	109375	EMX 1 Off Target Site # 2
Plasmid, BE3	85576	86600	169592	EMX 1 Off Target Site # 2
Plasmid, HF-BE3	140317	145738	137050	EMX 1 Off Target Site # 2
Control	84818	125139	85454	EMX 1 Off Target Site # 2
Protein, BE3	11940	36593	24946	EMX1 Off Target Site # 3
Protein, HF-BE3	26762	31566	36377	EMX1 Off Target Site # 3
Plasmid, BE3	32420	21547	14659	EMX1 Off Target Site # 3
Plasmid, HF-BE3	31427	16592	17385	EMX1 Off Target Site # 3
Control	17385	28128	32717	EMX1 Off Target Site # 3

Murine Samples from NIH 3T3 cell treatment

Sample Description	Replicate 1	Replicate 2	Replicate 3	Amplicon
Plasmid, BE3	16641	102216	46361	On Target VEGFA (Mus)
Plasmid, HF-BE3	89330	126545	100993	On Target VEGFA (Mus)
Protein, BE3	88998	81697	51124	On Target VEGFA (Mus)
Protein, HF-BE3	128218	29193	131515	On Target VEGFA (Mus)
Control	18767	38866	58985	On Target VEGFA (Mus)
Plasmid, BE3	174782	167504	182565	CFD Off Target 1
Plasmid, HF-BE3	167120	182520	192389	CFD Off Target 1
Protein, BE3	230569	212605	138144	CFD Off Target 1
Protein, HF-BE3	228668	211457	183370	CFD Off Target 1
Control	171738	191117	20879	CFD Off Target 1
Plasmid, BE3	206475	227332	206089	CFD Off Target 2
Plasmid, HF-BE3	213809	203028	199078	CFD Off Target 2
Protein, BE3	215995	275754	249969	CFD Off Target 2
Protein, HF-BE3	250918	272063	241059	CFD Off Target 2
Control	193760	175959	246963	CFD Off Target 2
Plasmid, BE3	60388	126278	7328	CFD Off Target 3
Plasmid, HF-BE3	89045	128508	5178	CFD Off Target 3
Protein, BE3	167195	330046	11163	CFD Off Target 3
Protein, HF-BE3	82120	309352	10393	CFD Off Target 3
Control	83204	176939	5661	CFD Off Target 3
Plasmid, BE3	192846	113709	171078	CFD Off Target 4
Plasmid, HF-BE3	205601	151434	188943	CFD Off Target 4
Protein, BE3	218194	181993	208398	CFD Off Target 4
Protein, HF-BE3	211966	148976	186838	CFD Off Target 4
Control	183933	130318	197476	CFD Off Target 4

Mouse Cochlea Samples

Sample Description	Replicate 1	Replicate 2	Replicate 3	Amplicon
Stria vasculari es	37889	205706	62091	On Target (VEGFA)
Organ of Corti	148447	175004	29075	On Target (VEGFA)
Modiolus	182806	181382	61269	On Target (VEGFA)
Uninjected control	228222	241979	272759	On Target (VEGFA)
Stria vascularies	44457	244487	244646	CFD Off Target 1
Organ of Corti	136335	118318	34747	CFD Off Target 1
Modiolus	67176	209543	68699	CFD Off Target 1
Uninjected control	343100	342717	379015	CFD Off Target 1
Stria vascularies	72962	319883	265793	CFD Off Target 2
Organ of Corti	198456	131430	60530	CFD Off Target 2
Modiolus	92014	251509	81413	CFD Off Target 2
Uninjected control	399138	345965	483920	CFD Off Target 2
Stria vascularies	8325	80322	142556	CFD Off Target 3
Organ of Corti	81014	45976	1810	CFD Off Target 3
Modiolus	9928	75555	11341	CFD Off Target 3
Uninjected control	399138	345965	483920	CFD Off Target 3
Stria vascularies	232194	397770	554054	CFD Off Target 4
Organ of Corti	313472	285302	176872	CFD Off Target 4
Modiolus	230105	371399	258142	CFD Off Target 4
Uninjected control	524503	637946	624709	CFD Off Target 4

Zebrafish samples

	1	2	3	Amplicon
Treated zebrafish	72355	49498	81061	TYR 1
Scrambled sgRNA	107919	98502	92429	TYR 1
Treated zebrafish	51434	48014	41547	TYR 2
Scrambled sgRNA	61466	62374	66765	TYR 2
Treated zebrafish	6487	57247	75883	TYR 3
Scrambled sgRNA	64596	71234	75624	TYR 3

Mouse Cochlea Samples - treated with unrealated sgRNA

	Sample: Stria	Sample: Cortii	Sample: Modiolus	Amplicon
	537459	249767	389274	On Target (VEGFA)

Supplementary Table 5: **Number of HTS reads that align to the reference sequence and pass the quality filters described in Materials and Methods.**

Supplementary References

1. Zuris, J.A. et al. Cationic lipid-mediated delivery of proteins enables efficient protein-based genome editing in vitro and in vivo. *Nature biotechnology* **33**, 73-80 (2015).
2. Tsai, S.Q. et al. GUIDE-seq enables genome-wide profiling of off-target cleavage by CRISPR-Cas nucleases. *Nature biotechnology* **33**, 187-197 (2015).
3. Doench, J.G. et al. Optimized sgRNA design to maximize activity and minimize off-target effects of CRISPR-Cas9. *Nature biotechnology* **34**, 184-191 (2016).
4. Haeussler, M. et al. Evaluation of off-target and on-target scoring algorithms and integration into the guide RNA selection tool CRISPOR. *Genome Biol* **17**, 148 (2016).
